# Supplementary material for: Meta-analysis of genome-wide association studies for body fat distribution in 694 649 individuals of European ancestry
Source: Hum Mol Genet. 2018 Sep 14;28(1):166–74. doi: 10.1093/hmg/ddy327 (PMC6298238; doi:10.1093/hmg/ddy327)
Supplement: Supplementary Data [file ddy327_supp.pdf]

Supplementary information for

Meta-analysis of genome-wide association studies for body fat distribution in 694,649 individuals of European ancestry

Sara L. Pulit[1,2,3*], Charli Stoneman[4*], Andrew P. Morris[5,6], Andrew R. Wood[4], Craig A. Glastonbury[1], Jessica Tyrrell[4], Loïc Yengo[7], Teresa Ferreira[1], Eirini Marouli[8], Yingjie Ji[4], Jian Yang[7,9], Samuel Jones[4], Robin Beaumont[4], Damien C. Croteau-Chonka[10], Thomas W. Winkler[11], GIANT Consortium, Andrew. T. Hattersley[4], Ruth J. F. Loos[12], Joel N. Hirschhorn[13,14,15,16], Peter M. Visscher[7,9], Timothy M. Frayling[4#], Hanieh Yaghootkar[4#], Cecilia M. Lindgren[1,3,6#]

Code and data release

For access to information related to this project, including code, downloadable supplemental tables, links to summary-level genome-wide association study data, and other supporting data for the project, please see the following GitHub repository: https://github.com/lindgrengroup/fatdistnGWAS.

More information is provided in the **'Code and Data Release'** section of this Supplementary Information.

Commonly-used abbreviations

(1) BMI: body mass index, measure of overall adiposity
(2) WHR: waist-to-hip ratio, measure of fat distribution
(3) WHRadjBMI: waist-to-hip ratio adjusted for BMI, measure of fat distribution independent of overall adiposity

Table of Contents

Supplementary Figures	Pages 03 - 36
Supplementary Tables	Pages 37 - 51
Code and Data Release	Pages 52 - 54
Supplementary Methods and Results	Pages 55 - 57
References	Pages 58 - 59

Supplementary Figures

Supplementary Figure 1 | Manhattan and QQ plots for meta-analysis of fat distribution and obesity phenotypes. We performed meta-analysis of our UK Biobank GWAS with existing GWAS data generated by the GIANT consortium. Manhattan and QQ plots from these meta-analyses in the combined sample for waist-to-hip ratio (max N = 697,734), waist-to-hip ratio adjusted for BMI (max N = 694,649) and BMI (max N = 806,834) are shown here. Note that the y-axes are not continuous and are disrupted at $p < 1 \times 10^{-100}$ or $p < 1 \times 10^{-50}$. Genome-wide significance was set at $p < 5 \times 10^{-9}$ to reflect the SNP density of the UK Biobank data [1]. Traditional genome-wide significance ($p < 5 \times 10^{-8}$) is indicated by the second, lower horizontal line.

a. Analysis of body mass index

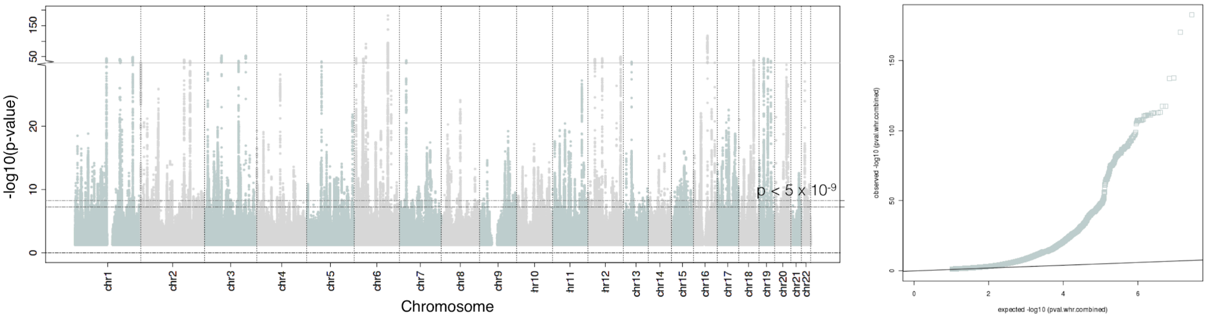

b. Analysis of waist-to-hip ratio

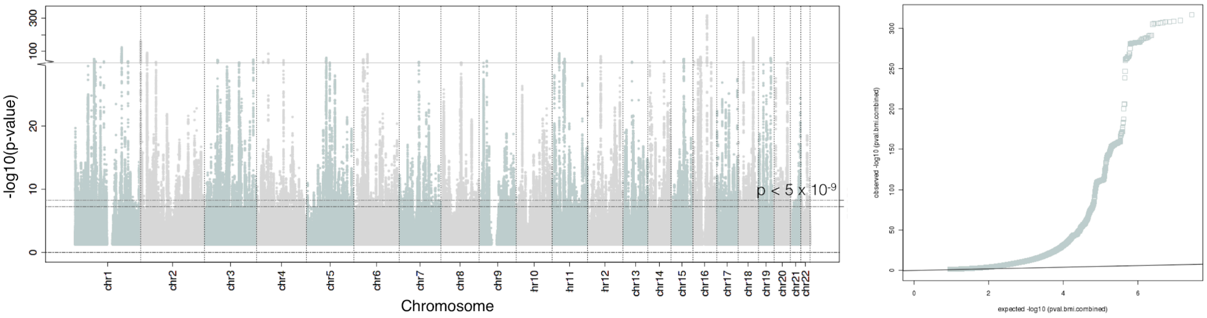

c. Analysis of waist-to-hip ratio adjusted for body mass index

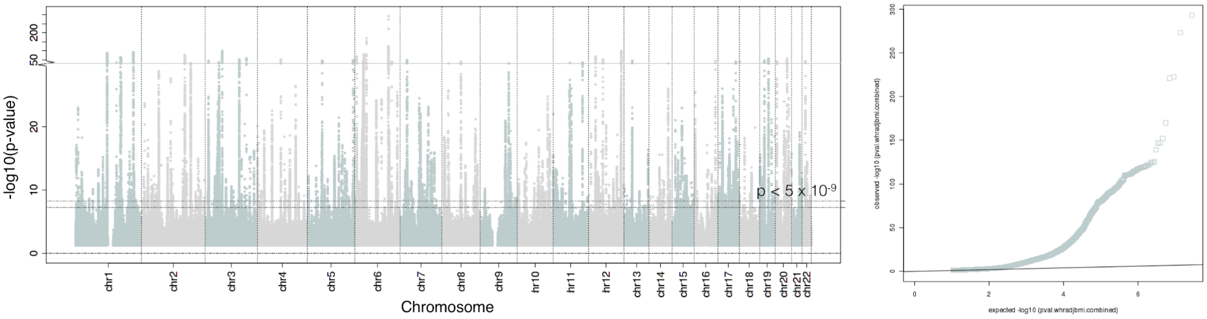

Supplementary Figure 2 | Comparison of index and secondary SNPs in sensitivity analysis GWAS in UK Biobank. To test the robustness of the primary (index) and secondary signals in our meta-analyses, we performed two GWAS sensitivity analyses in UK Biobank: (1) in unrelated samples only, and (2) in unrelated white British samples only. We meta-analyzed the results with the previous GWAS in obesity and fat distribution from GIANT [2,3], compared the summary statistics across the three meta-analyses, and found correlation (Pearson's r, shown on plots) to be strong. Where we observed weaker correlations, we found that a very low-frequency SNP in the (smaller) white British analysis was the cause; excluding SNPs with frequency < 0.1% yielded high correlations (Pearson's r > 0.95) across all metrics.

a. Body mass index signals in the original meta-analysis (all samples; x-axis) and the sensitivity meta-analyses (y-axis)

Combined samples

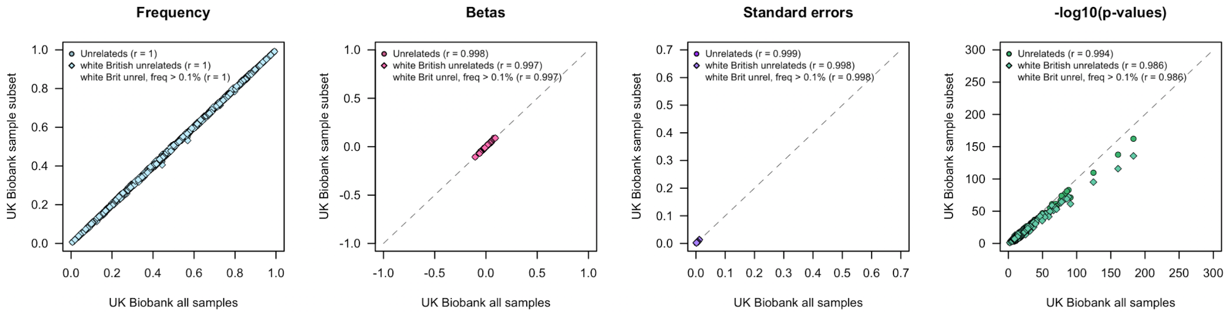

Women only

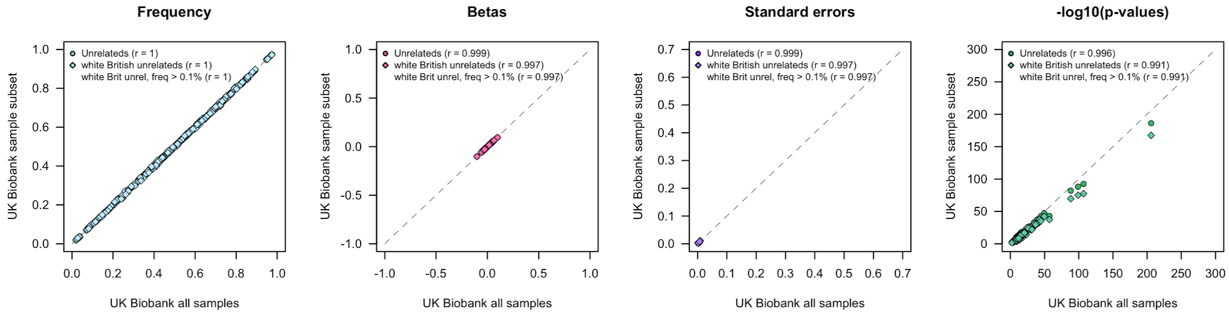

Men only

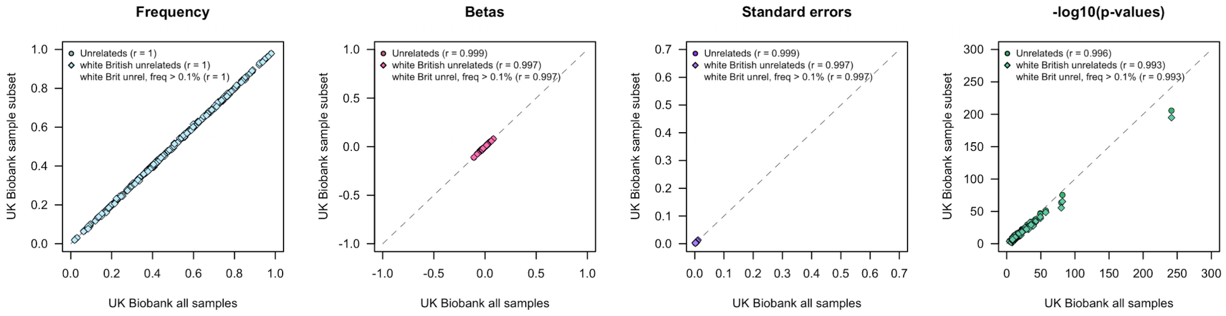

b. Waist-to-hip ratio signals in the original meta-analysis (all samples; x-axis) and the sensitivity meta-analyses (y-axis)

Combined samples

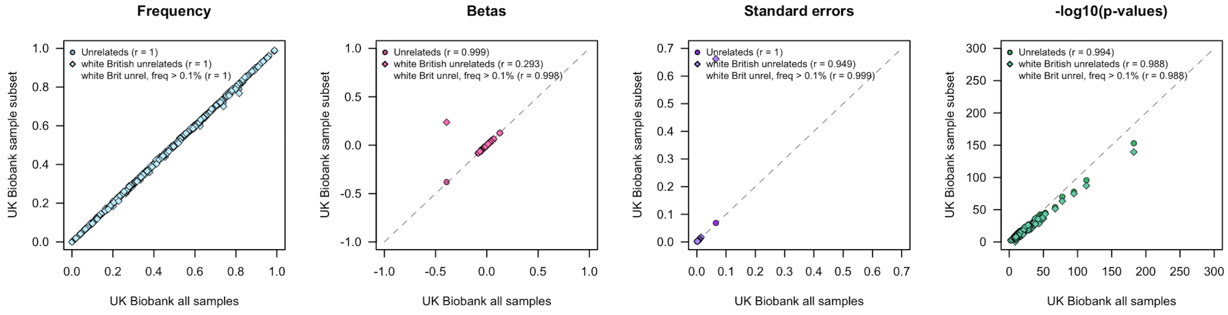

Women only

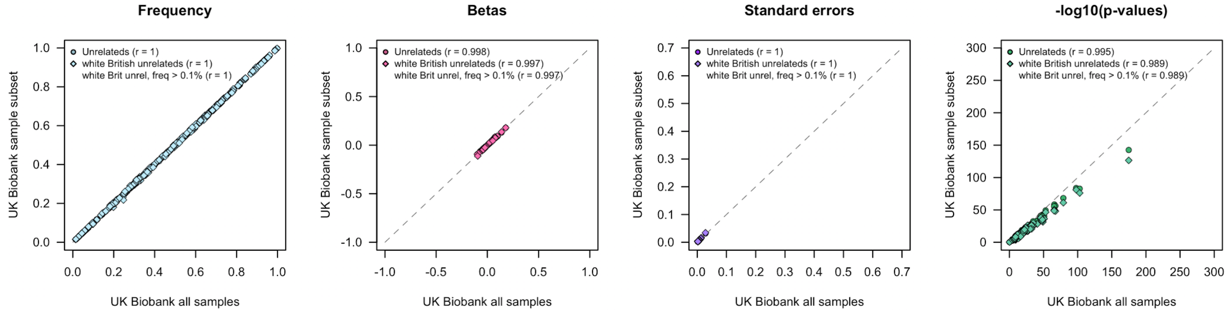

Men only

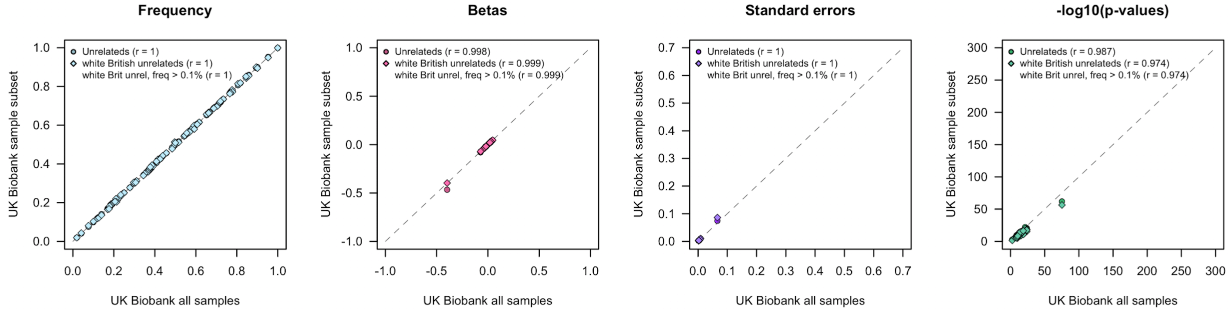

c. Waist-to-hip ratio adjusted for BMI signals in the original meta-analysis (all samples; x-axis) and the sensitivity meta-analyses (y-axis)

Combined samples

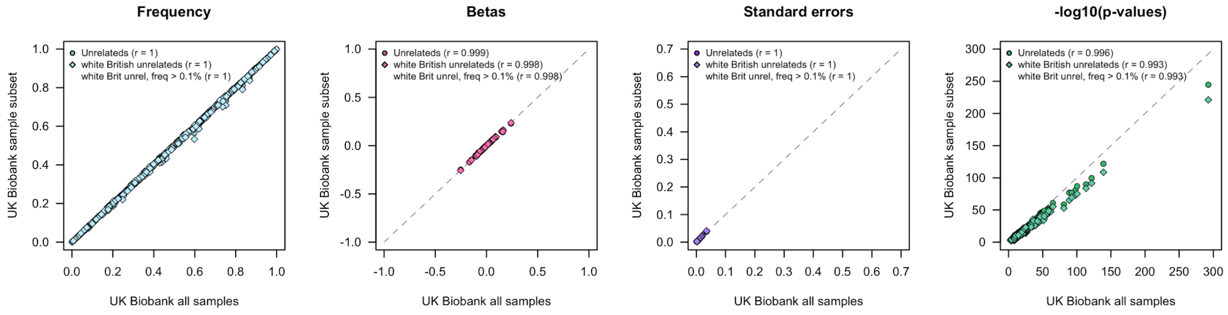

Women only

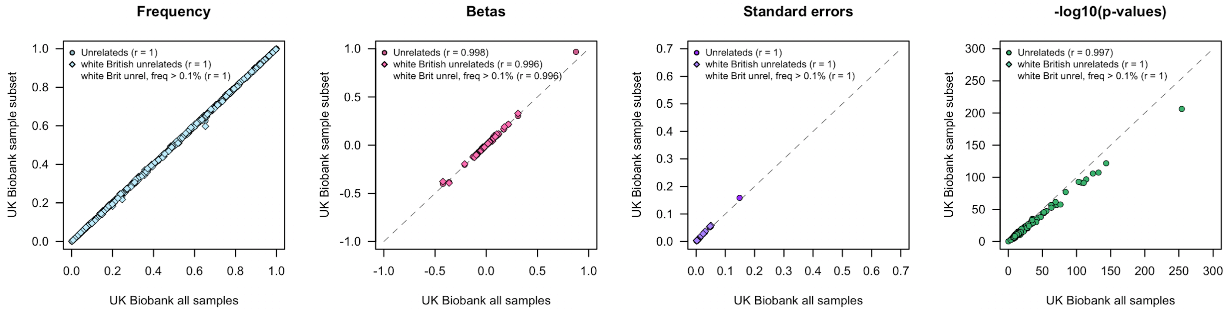

Men only

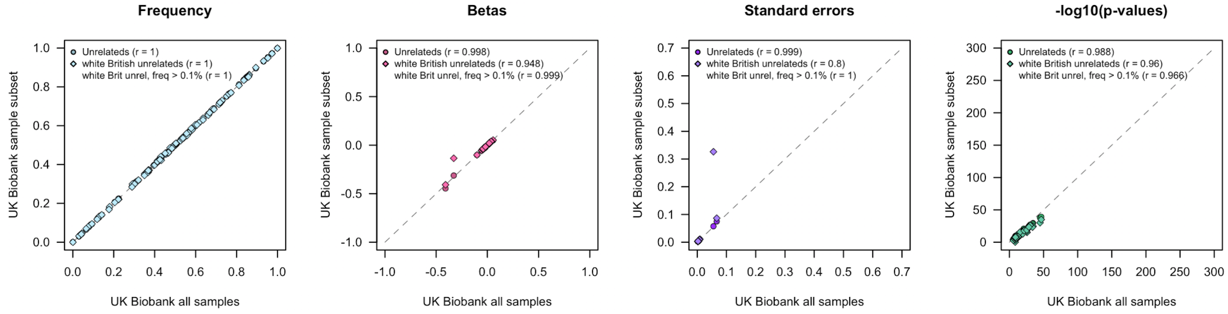

Supplementary Figure 3 | Comparison of SNPs in sensitivity analysis of GIANT population-based studies and UK Biobank meta-analyses. To evaluate whether dichotomizing samples into cases and controls introduced any bias in the GIANT meta-analysis of waist-hip ratio adjusted for BMI (WHRadjBMI), we meta-analysed population-based studies from GIANT [3] (73,925 individuals from 29 studies) with UK Biobank study (N = 449,216) (**Supplementary Table 15)**. We compared SNPs reaching genome-wide significance, using traditional genome-wide significance threshold ($p < 5 \times 10^{-8}$), that were present in all four branches of the two meta-analyses: all GIANT studies from the original meta-analysis, UK Biobank from the original meta-analysis, GIANT population-based only studies from the sensitivity meta-analysis and UK Biobank from the sensitivity meta-analysis. We found strong correlations between the two meta-analysis results (Pearson's r, shown on plots) indicating that dichotomizing samples into cases and controls introduced very limited bias.

The 29 studies included are: the Atherosclerosis Risk in Communities study (ARIC), British 1958 Birth Cohort (B58C)-T1DGC, Family Heart Study (FamHS), Croatia, Framington Heart Study (FHS), Erasmus Rucphen Family (ERF), KORAS3, ORKNEY, Rotterdam Study Base 1 (RS1), Sardinia, Study of Health in Pomerania (SHIP), Estonian Genome Center of University of Tartu (EGCUT), Northern Finland Birth Cohort (NFBC), B58C-WTCCC, CoLaus, The European Prospective Investigation into Cancer and Nutrition (EPIC), TwinsUK, KORAS4, Helsinki Birth Cohort Study (HBCS), InChianti, LifeLines, The London Life Sciences Prospective Population Study (LOLIPOP) EW610, Tracking Adolescents' Individual Lives Survey (TRAILS), SHIP-Trend, EGCUT-370, EGCUT-OMNI, Rotterdam Study Base 2 (RS2), Rotterdam Study Base 3 (RS3) and HERITAGE. See 'Code and Data Release' for links to the GIANT summary-level data.

a. Waist-to-hip ratio adjusted for BMI genome-wide significant signals ($P < 5 \times 10^{-8}$) in the original meta-analysis (GIANT all & UK Biobank; x-axis) and the sensitivity meta-analysis (GIANT population-based only & UK Biobank; y-axis)

Combined Samples

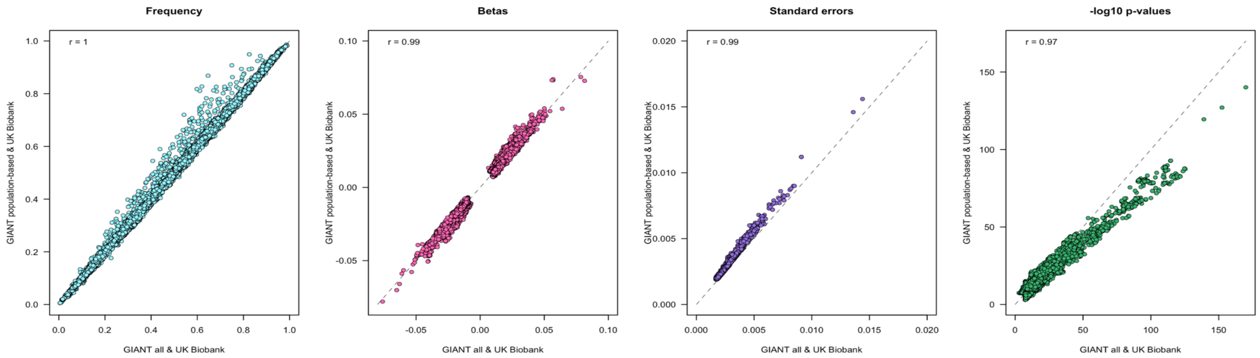

Women only

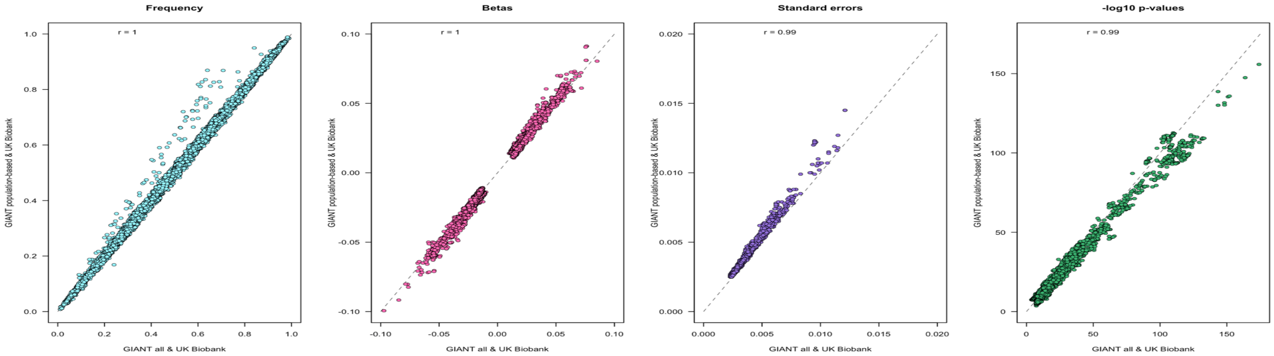

Men only

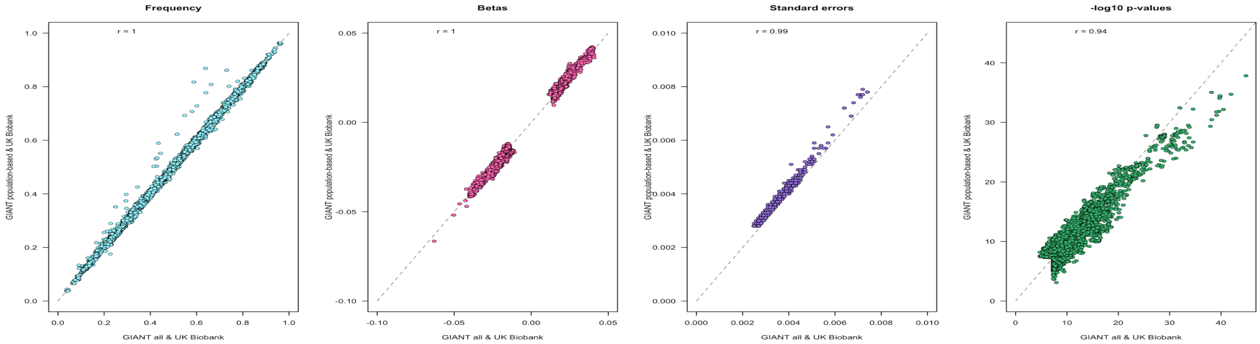

b. Waist-to-hip ratio genome-wide significant signals ($P < 5 \times 10^{-8}$) in the original meta-analysis (GIANT all & UK Biobank; x-axis) and the sensitivity meta-analysis (GIANT population-based only & UK Biobank; y-axis)

Combined Samples

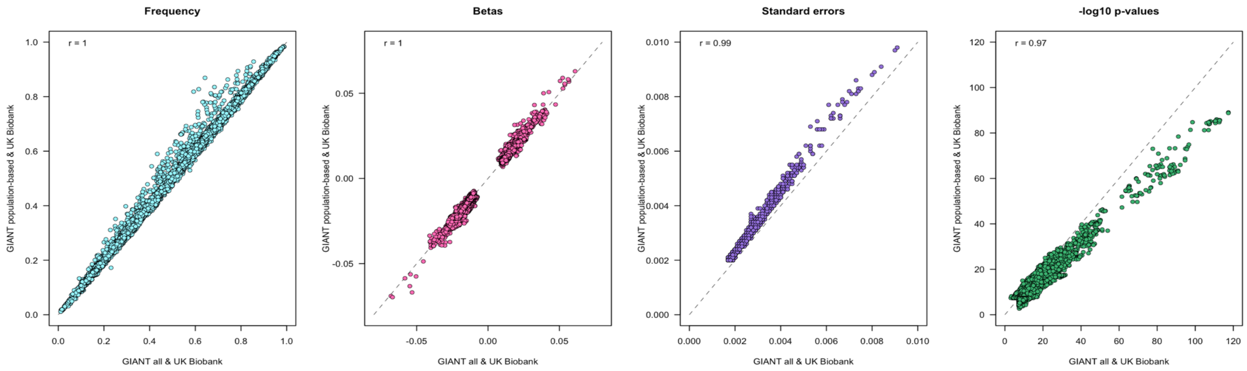

Women only

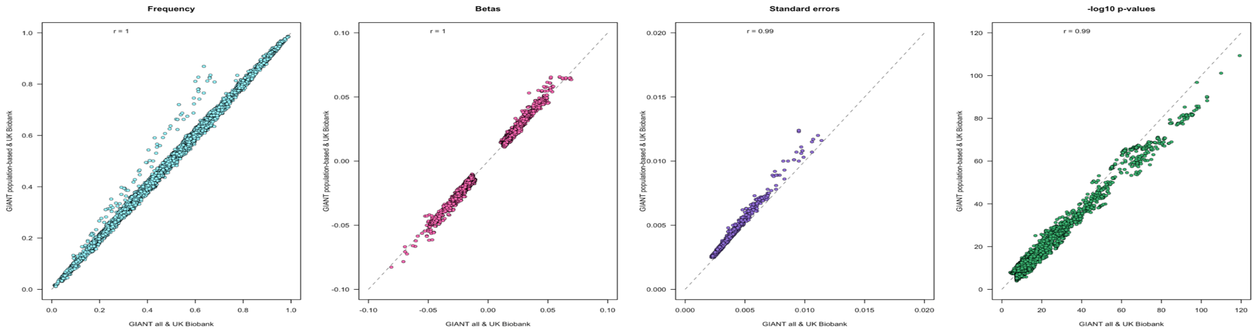

Men only

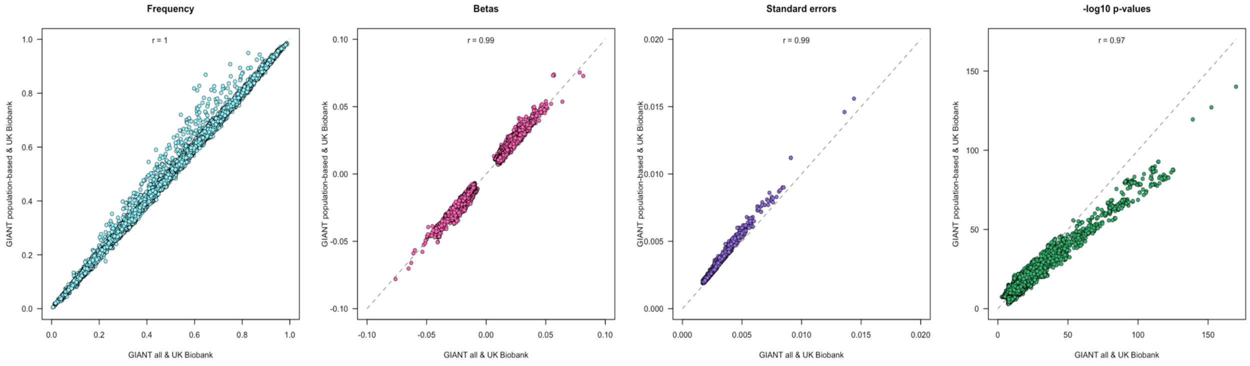

c. Waist-to-hip ratio adjusted for BMI genome-wide significant signals ($P < 5 \times 10^{-8}$) in the original meta-analysis (GIANT all & UK Biobank; x-axis) and the sensitivity meta-analysis (GIANT population-based only & UK Biobank; y-axis)

Combined Samples

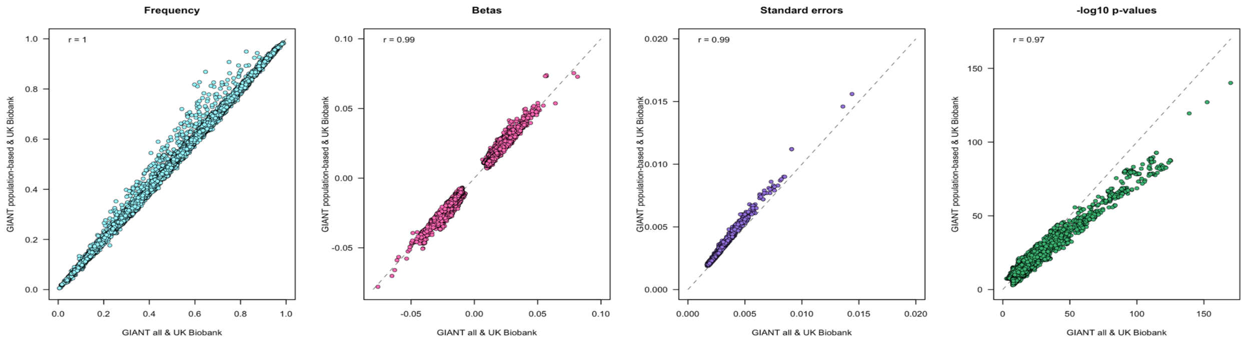

Women only

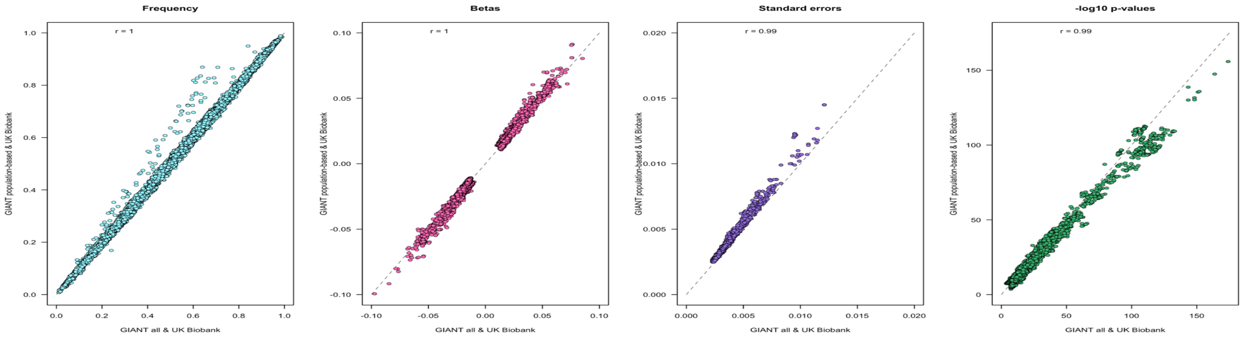

Men only

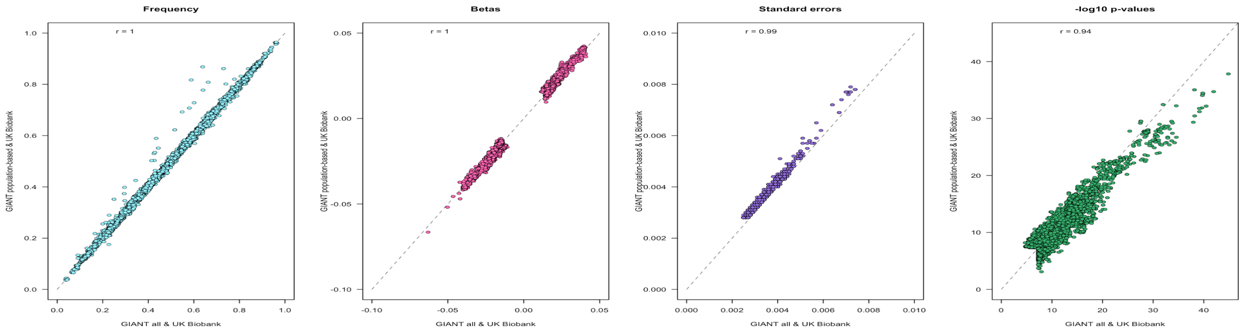

d. Waist-to-hip ratio genome-wide significant signals ($P < 5 \times 10^{-8}$) in the original meta-analysis (GIANT all & UK Biobank; x-axis) and the sensitivity meta-analysis (GIANT population-based only & UK Biobank; y-axis)

Combined Samples

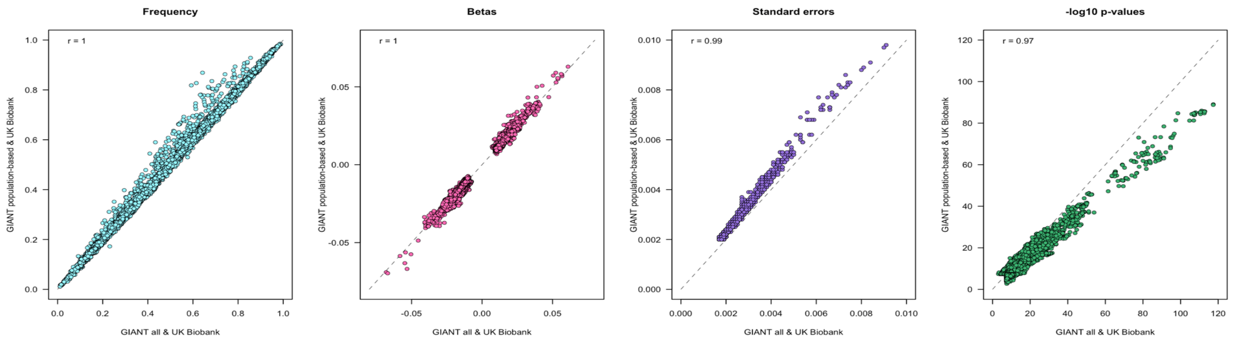

Women only

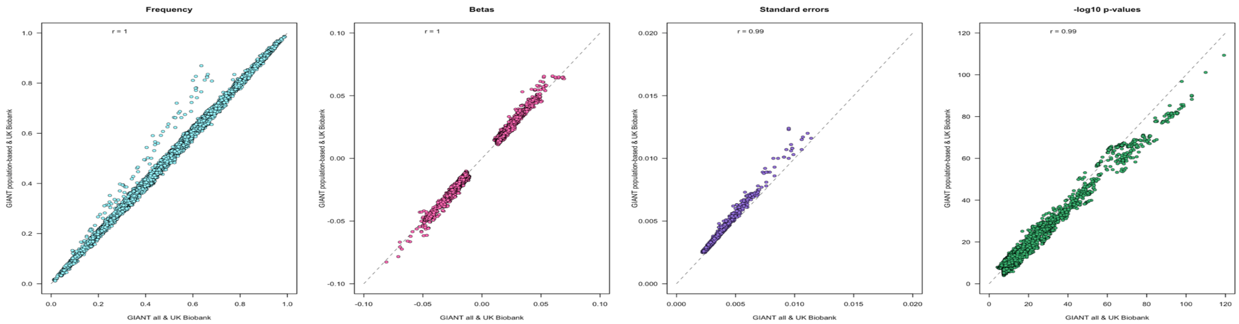

Men only

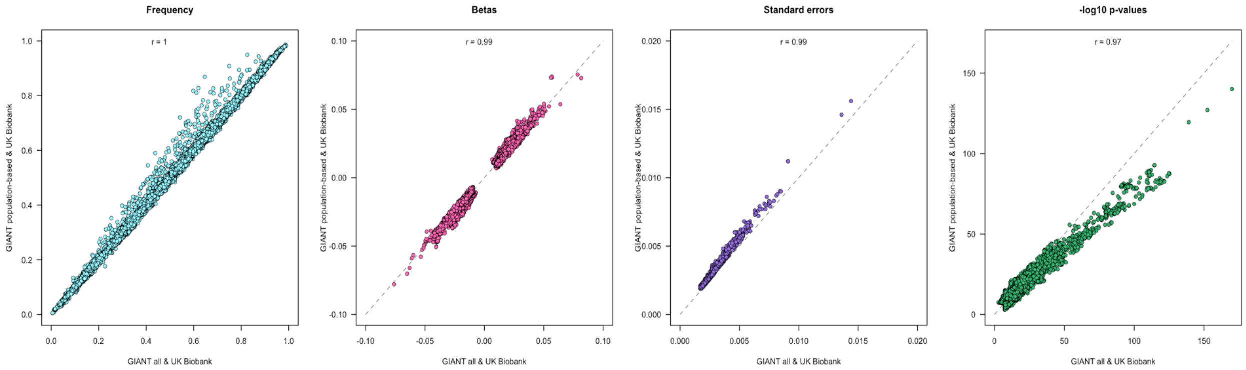

Supplementary Figure 4 | Test of collider bias at genome-wide associated SNPs. Conditioning a variable on a second, correlated variable (sometimes called conditioning on a 'collider') can induce both false-positive and false-negative associations [4,5]. Body mass index (BMI) and waist-to-hip ratio (WHR) correlate to one another (the correlation between 2 traits after correcting for age, sex, PCs, centres and genotype chip is 0.5 in 378,178 unrelated European from UK Biobank study; p = 2 x 10^{-16}); therefore, conditioning WHR on BMI to generate the waist-to-hip ratio adjusted for BMI (WHRadjBMI) phenotype may have resulted in collider bias at genome-wide associated SNPs.

We examined the association statistics of WHRadjBMI index SNPs in meta-analyses of BMI and WHR (see Supplementary Methods). WHRadjBMI-associated SNPs that show a stronger association with BMI than with WHR (green points) potentially suffer from collider bias. SNPs with an association >2 orders of magnitude stronger in BMI than in WHR (blue-green points) show stronger effects of collider bias. Consistently, these tend to be SNPs with near-zero effects in WHR and non-zero effects in BMI (righthand panels). The data underlying these figures are provided in Supplementary Table 2.

a. Collider bias analysis in 346 index SNPs from the combined sample analysis

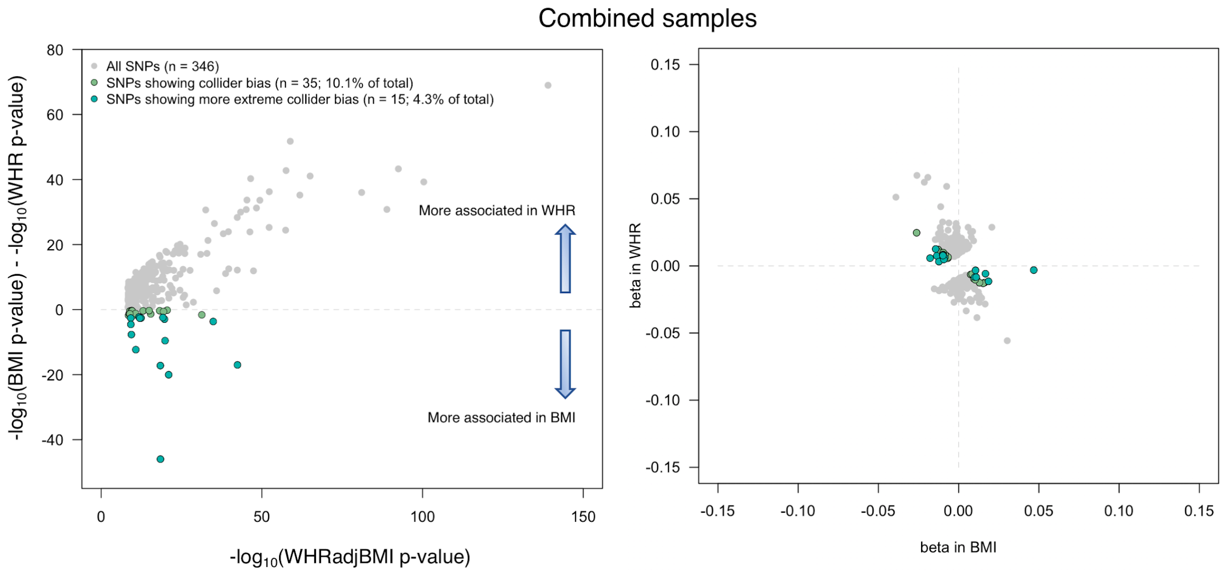

b. Collider bias analysis in 346 index SNPs from the women-only analysis

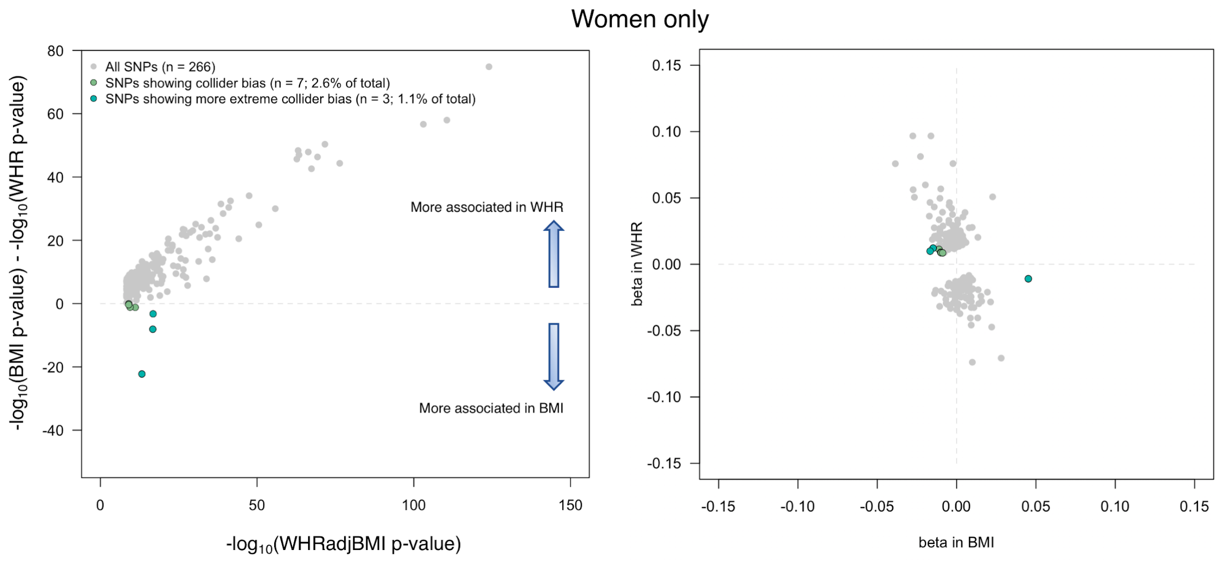

c. Collider bias analysis in 346 index SNPs from the men-only analysis

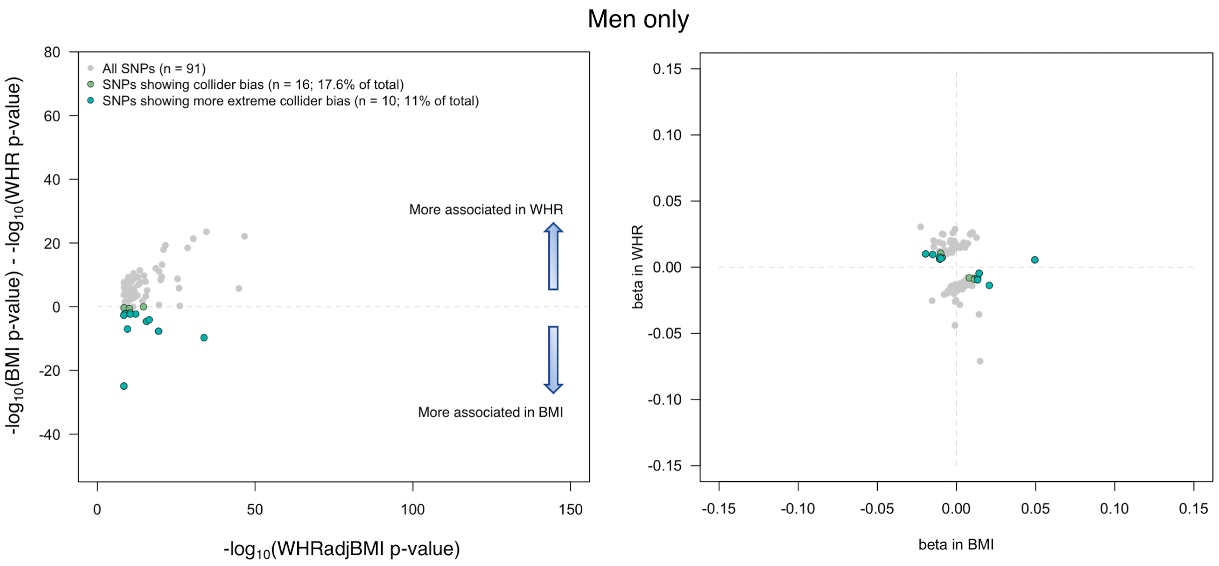

Supplementary Figure 5 | Miami and QQ plots for sex-specific meta-analyses of fat distribution and obesity phenotypes. Shown are sex-stratified results from meta-analyses for waist-to-hip ratio, waist-to-hip ratio adjusted for BMI and BMI. Note that the y-axes are not continuous and are disrupted at $p < 1 \times 10^{-100}$ or $p < 1 \times 10^{-50}$. Genome-wide significance was set at $p < 5 \times 10^{-9}$ to reflect the SNP density of the UK Biobank data [1]. Traditional genome-wide significance ($p < 5 \times 10^{-8}$) is indicated by the second, lower horizontal line.

a. Analysis of body mass index (max $N_{females}$ = 434,794 and max N_{males} = 374,756)

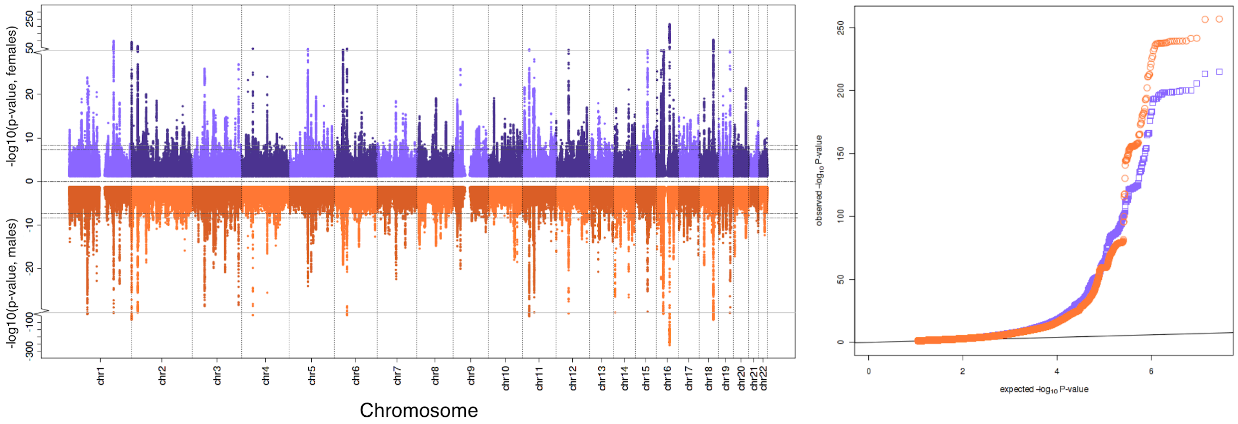

b. Analysis of waist-to-hip ratio (max $N_{females}$ = 381,152 and max N_{males} = 316,772)

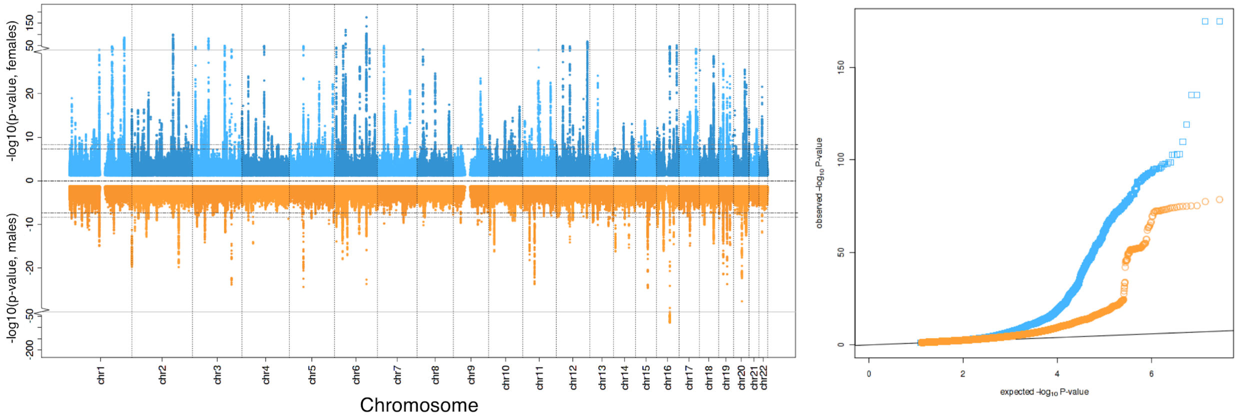

c. Analysis of waist-to-hip ratio adjusted for body mass index (max $N_{females}$ = 379,501 and max N_{males} = 315,284)

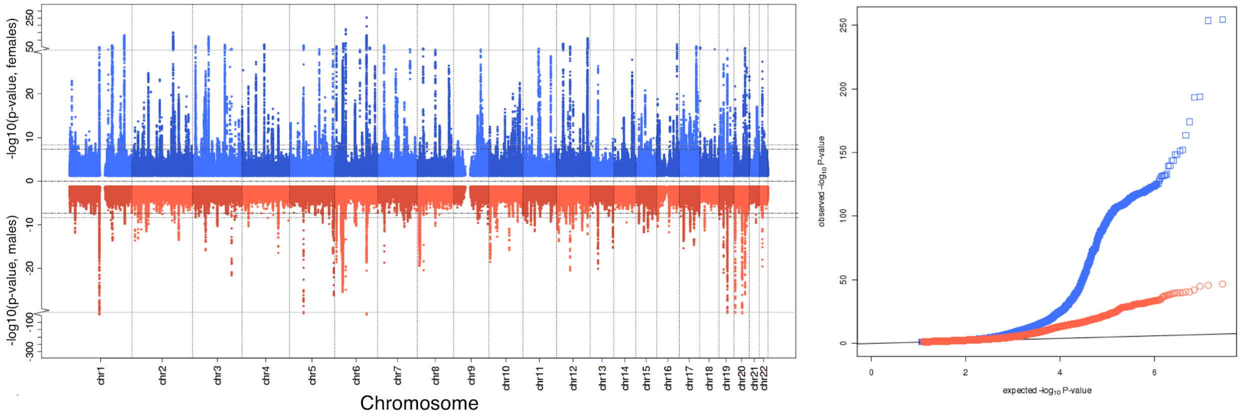

Supplementary Figure 6 | Test for sex-dimorphism of index SNPs from genome-wide significant loci. For each locus revealed in each of our meta-analyses (combined samples and sex-specific analyses), we tested for evidence of sex-dimorphism at the index SNP. We repeated these analyses for all phenotypes (waist-to-hip ratio, waist-to-hip ratio adjusted for BMI, and BMI). Approximately 27% of the SNPs associated to waist-to-hip ratio adjusted for BMI (discovered in the sex-specific analyses) and approximately 24% of the SNPs associated to waist-to-hip ratio (discovered in the sex-specific analyses) show evidence of sex-dimorphism. None of the loci discovered through sex-specific analysis of BMI were sex-dimorphic. A full table of the sex-dimorphic SNPs appears in **Supplementary Table 1**. Non-significant points are shown in faded colors, and points are sized by the $-\log^{10}(p_{diff})$ test for sex-dimorphism. Horizontal bars indicate standard error in men; vertical bars indicate standard error in women.

a. Index SNPs from combined and sex-specific analyses of waist-to-hip ratio.

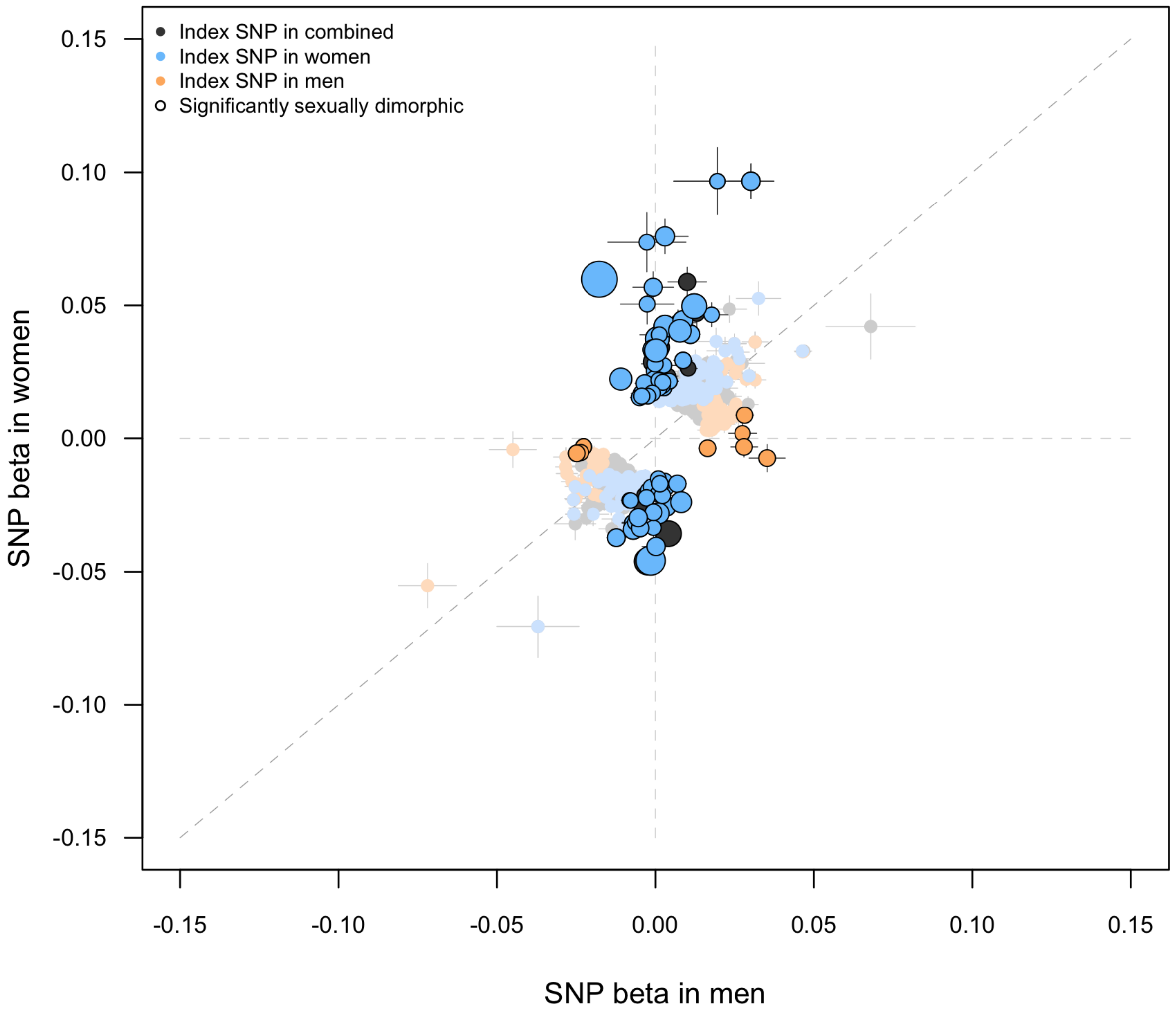

b. Index SNPs from combined and sex-specific analyses of body mass index.

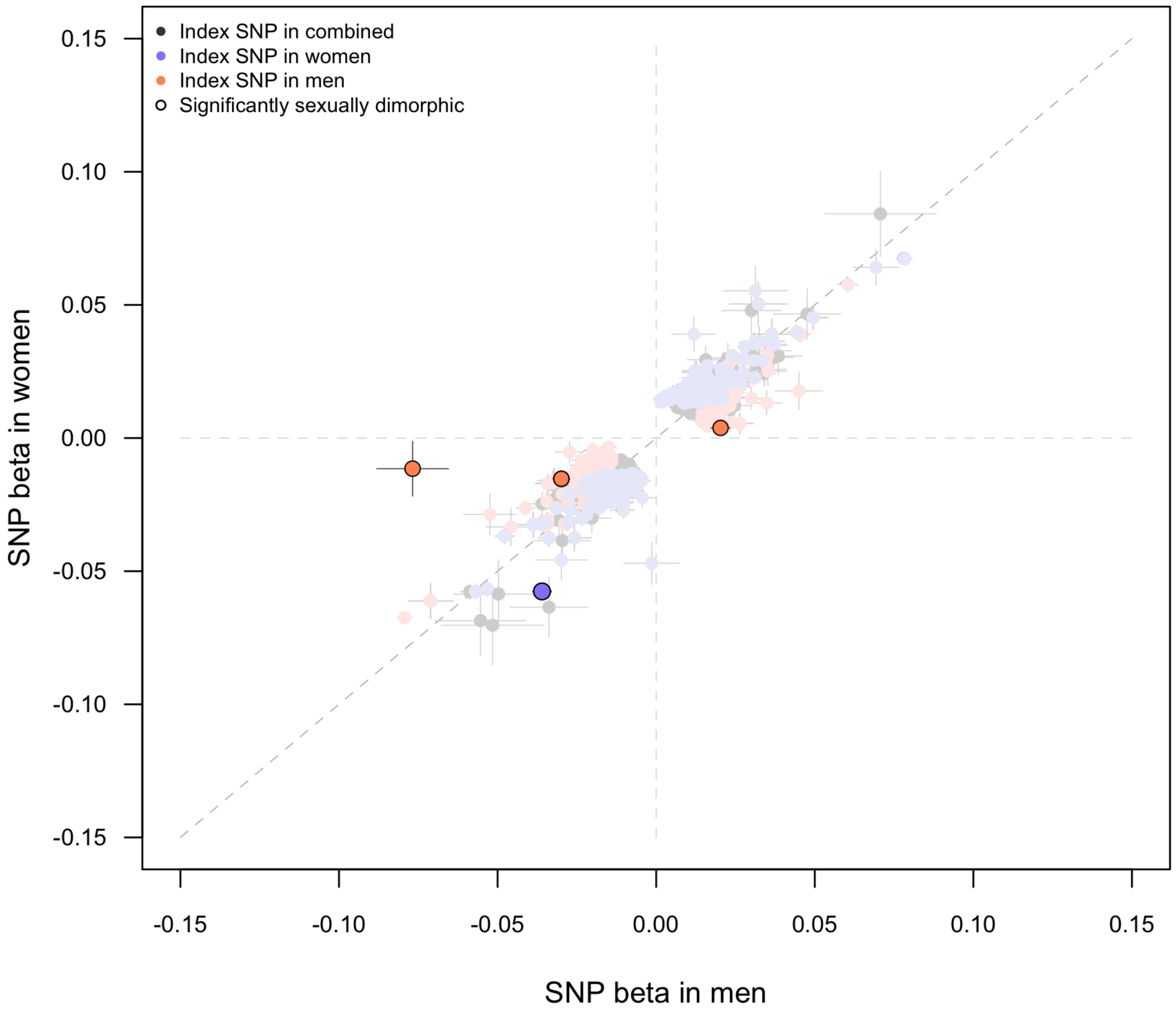

Supplementary Figure 7 | The effect of the 105 sex-specific WHRadjBMI SNPs on body fat percentage in men and women. We further investigated the effect of 97 female-specific and 8 male-specific WHRadjBMI SNPs on body fat percentage (BF%) in men and women. The 97 female-specific WHRadjBMI SNPs appear to have little effect on male WHRadjBMI (a) but have a similar effect on male and female BF% (b). Variants that have a female-specific effect on body fat distribution have a body fat phenotype in men and women. Of the 97 female-specific SNPs, 28 are associated with BF% in males ($p < 0.05/105 = 4.8 \times 10^{-4}$) and 25 are associated with BF% in females ($p < 0.05/105 = 4.8 \times 10^{-4}$). Of the 8 male-specific SNPs, 2 are associated with BF% in females ($p < 0.05/105 = 4.8 \times 10^{-4}$) and 3 ($p < 0.05/105 = 4.8 \times 10^{-4}$) are associated with BF% in males. Horizontal and vertical bars in each plot represent the 95% confidence intervals and points coloured in red signify a strong association with BF% (Bonferroni corrected $p < 0.05/105 = 4.8 \times 10^{-4}$). Note: rs547943994 has been excluded from the plots as this SNP was missing in the analysis of BF% in the UK Biobank.

a. Comparison of the effect of 97 female-specific SNPs on WHRadjBMI in males and females. The female-specific WHRadjBMI have very little effect on male WHRadjBMI.

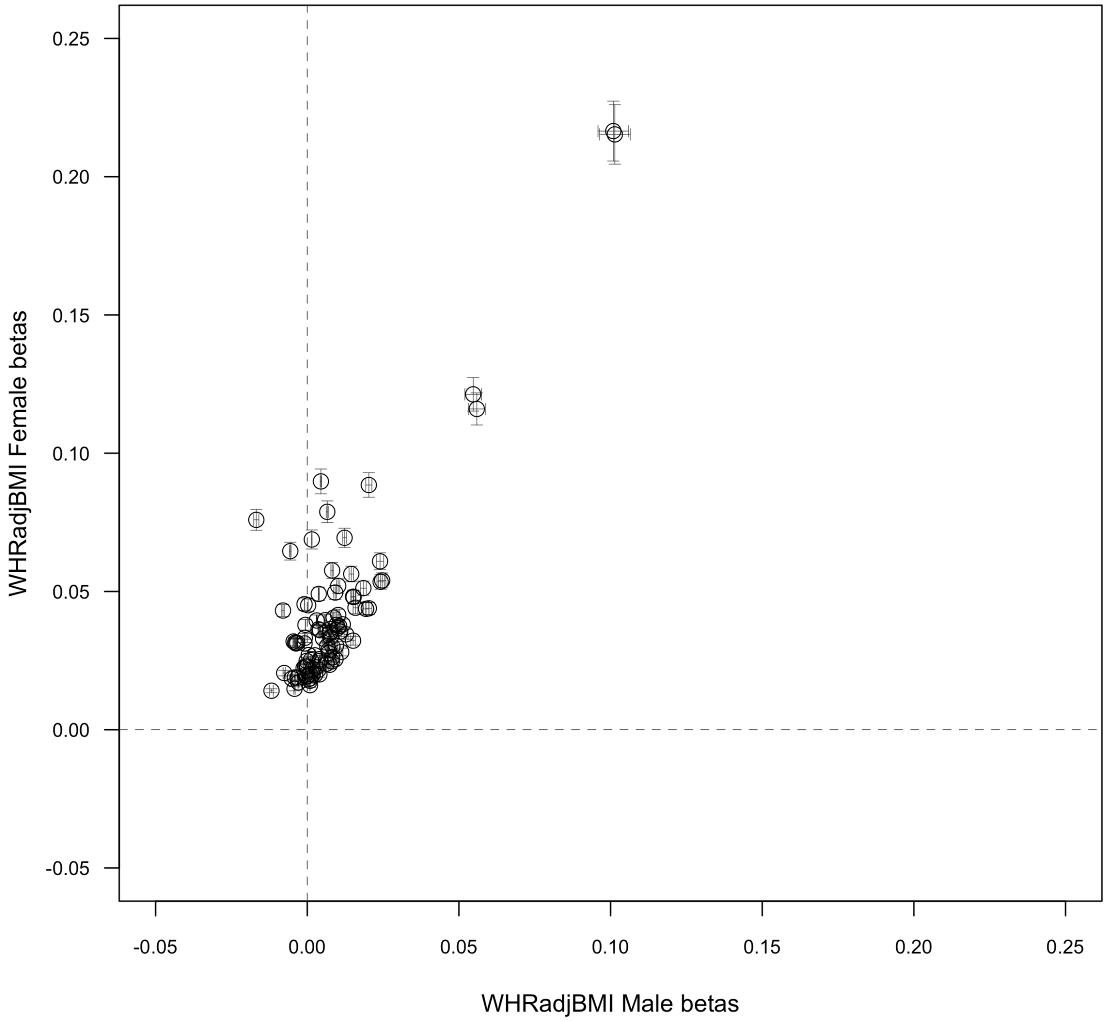

b. Comparison of the effect of 97 female-specific WHRadjBMI SNPs on BF% in males and females. The female-specific WHRadjBMI-increasing alleles have a similar effect on BF% in males and females. 28 of the female-specific SNPs are strongly associated with BF% in males whilst only 25 are strongly with BF% in females ($p < 0.05/105 = 4.8 \times 10^{-4}$)

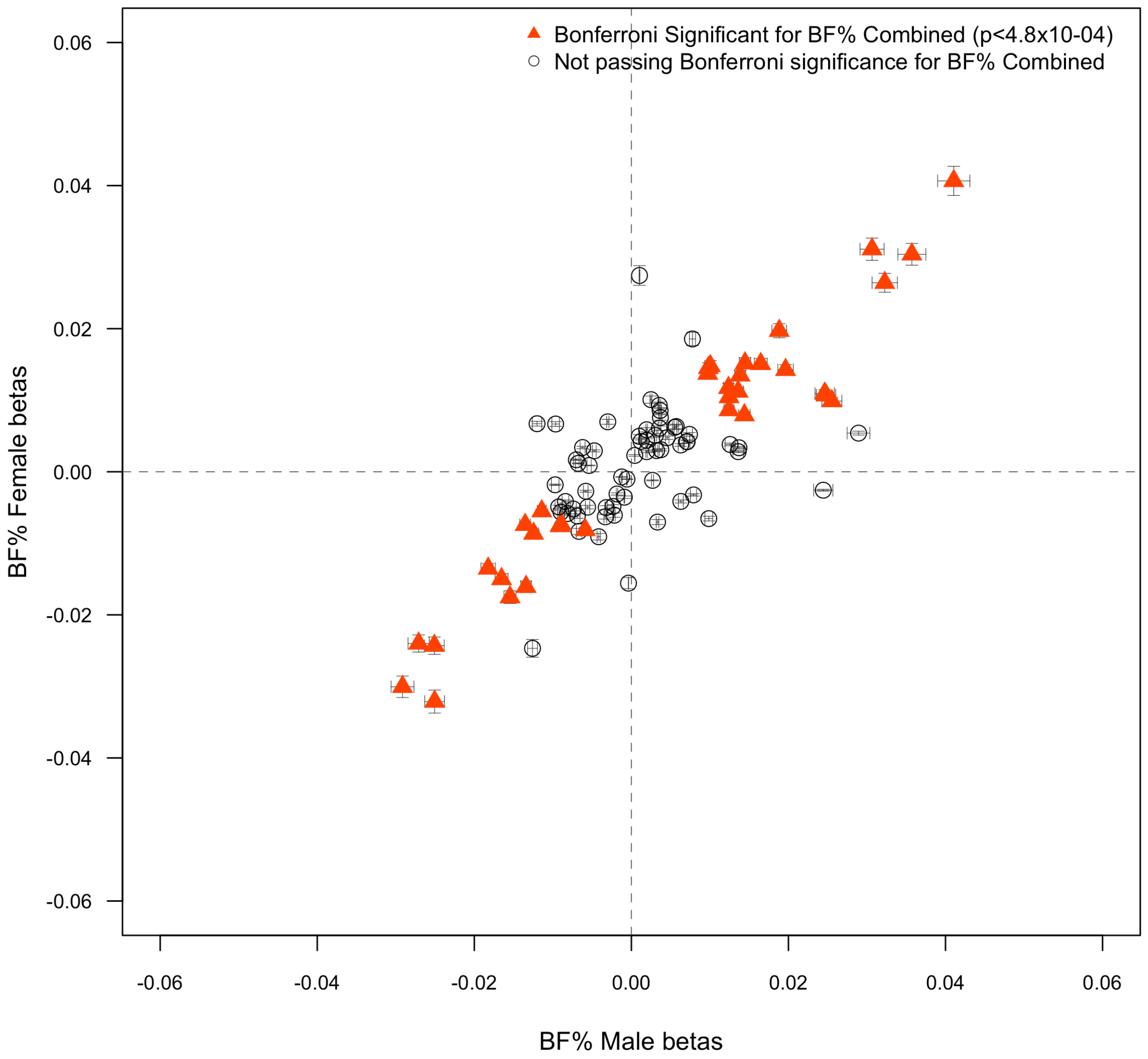

c. Comparison of the effect of 8 male-specific WHRadjBMI SNPs on WHRadjBMI in males and females. Four of the male-specific WHRadjBMI SNP have similar effect on WHRadjBMI in males and females whilst the other 4 male-specific WHRadjBMI SNPs have paradoxical effect on WHRadjBMI in males and females.

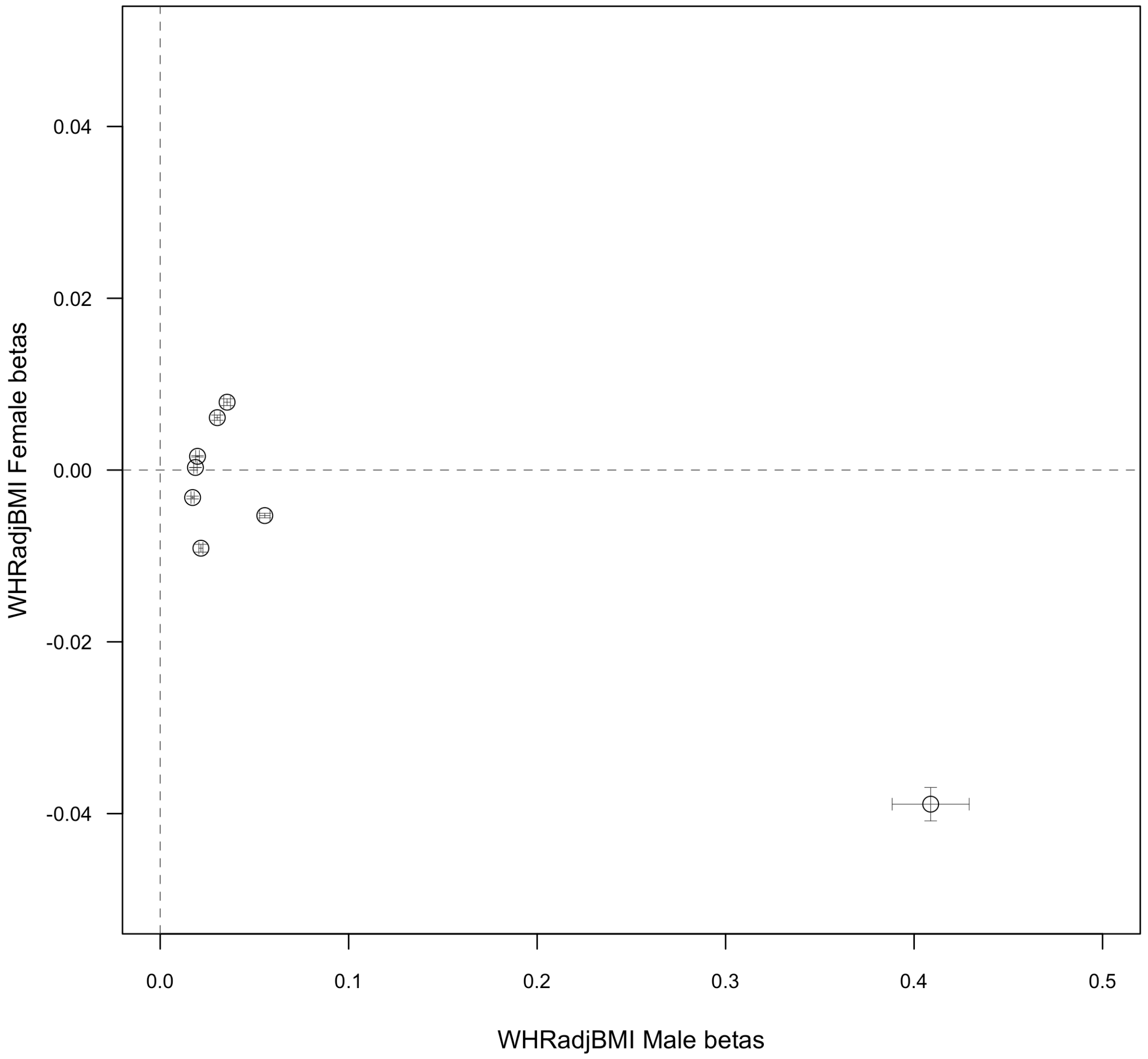

d. Comparison of the effect of 8 male-specific WHRadjBMI SNPs on BF% in males and females. The male-specific WHRadjBMI increasing alleles have a heterogeneous effect on BF% in males and females. 3 of the male-specific SNPs are strongly associated with BF% in males and 2 are strongly associated with BF% in females ($p < 0.05/105 = 4.8 \times 10^{-4}$).

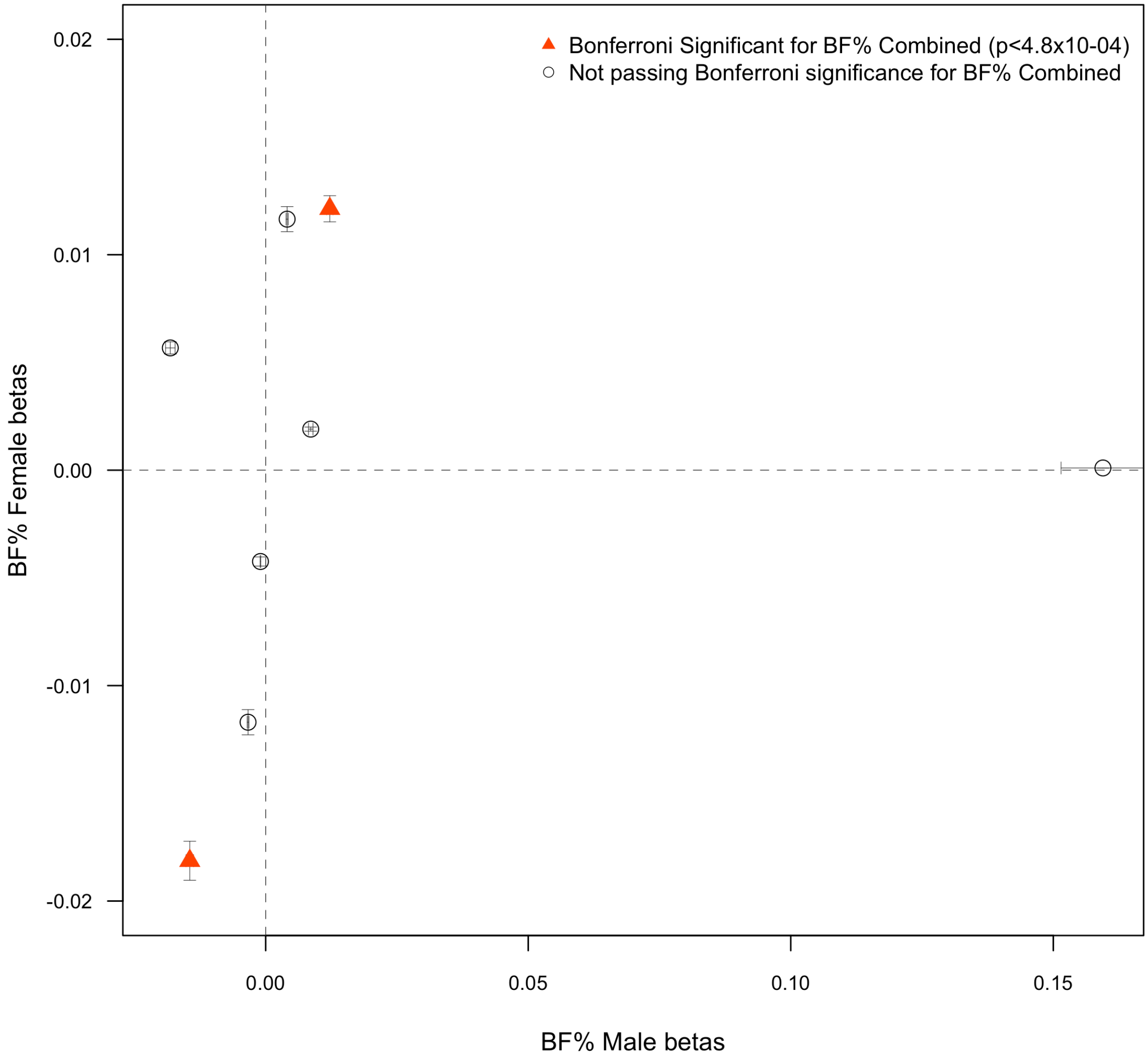

Supplementary Figure 8 | Concordance between WHRadjBMI-associated SNPs and SNPs from a genome-wide association study of imaging-based measures of subcutaneous and ectopic fat. Recently, Chu et al [6] performed a genome-wide association study in a multi-ancestry sample, examining 9 different subcutaneous and ectopic fat depots. We downloaded the summary-level data (see Code and Data Release) and examined the effect of WHRadjBMI-associated SNPs on different measures of fat depots from the Chu et al GWAS. Adjusting for 3 sample groups (combined, females only, and males only) and 8 phenotypes (the 8 fat depots) we found a strong correlation between alleles associated with higher WHRadjBMI and higher PAT, higher VAT, higher VATSAT and lower SAT. The effect of WHRadjBMI index SNPs from female analysis on measures of fat depots was stronger than the effect of index SNPs from male analysis.

Subcutaneous adipose tissue, SAT; visceral adipose tissue, VAT; pericardial adipose tissue (PAT); height, Ht; weight, Wt; body mass index, BMI; SAT Hounsfield units, SATHU; VAT Hounsfield units, VATHU; ratio of VAT and SAT, VATSAT.

Combined samples

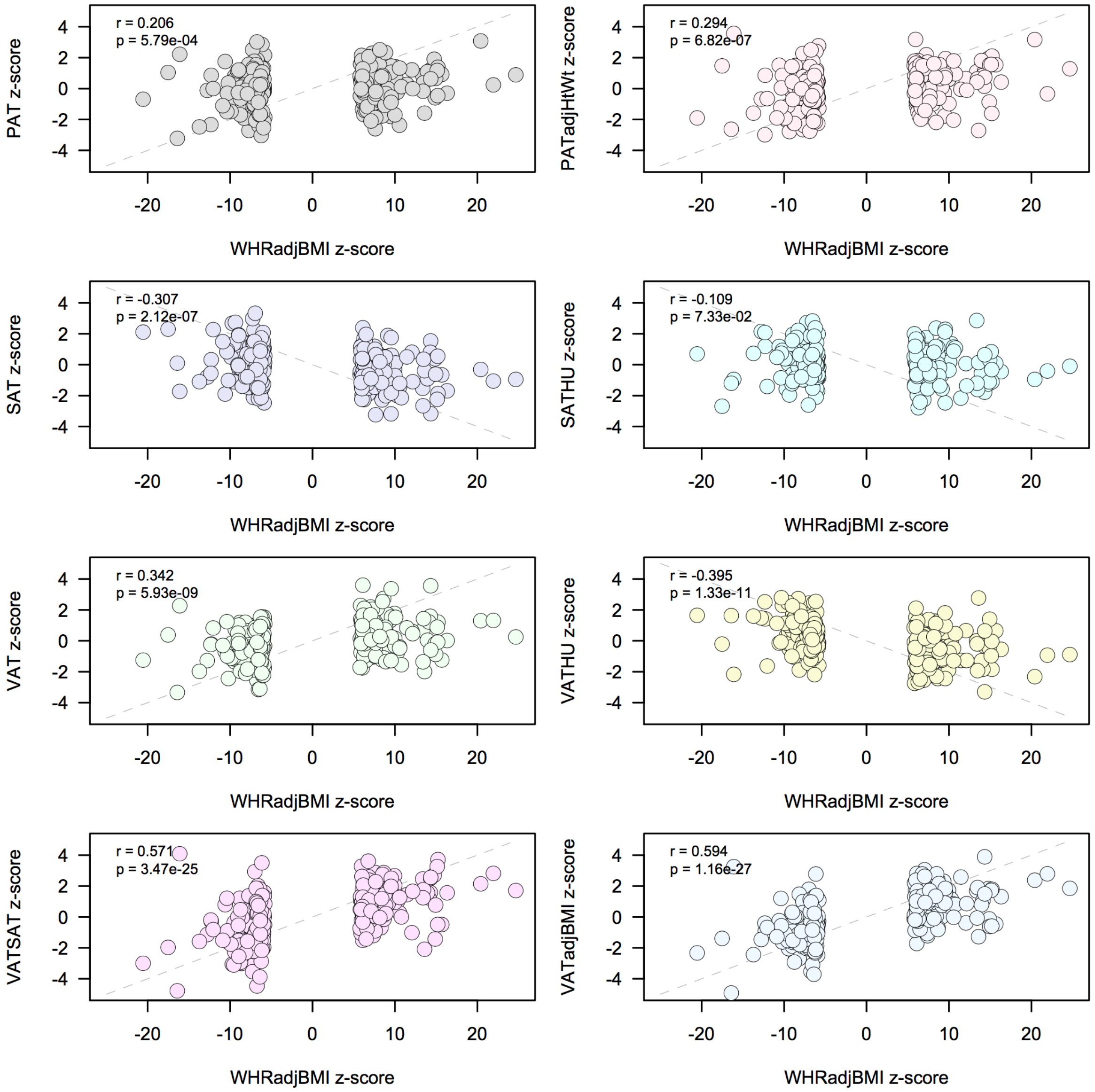

Women only

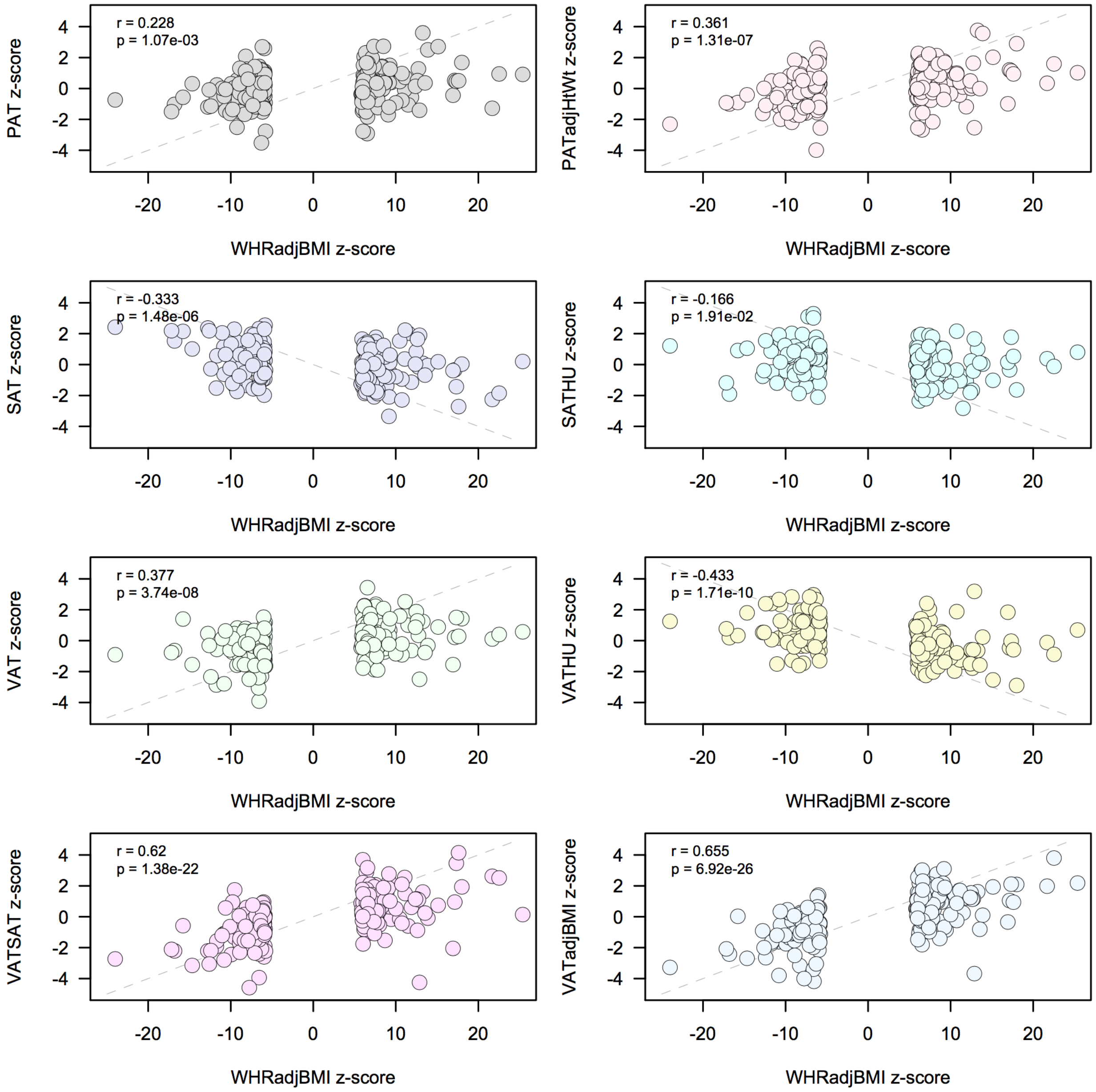

r = 0.228
p = 1.07e-03
PAT z-score
WHRadjBMI z-score
r = 0.361
p = 1.31e-07
PATadjHtWt z-score
WHRadjBMI z-score
r = -0.333
p = 1.48e-06
SAT z-score
WHRadjBMI z-score
r = -0.166
p = 1.91e-02
SATHU z-score
WHRadjBMI z-score
r = 0.377
p = 3.74e-08
VAT z-score
WHRadjBMI z-score
r = -0.433
p = 1.71e-10
VATHU z-score
WHRadjBMI z-score
r = 0.62
p = 1.38e-22
VATSAT z-score
WHRadjBMI z-score
r = 0.655
p = 6.92e-26
VATadjBMI z-score
WHRadjBMI z-score

Men only

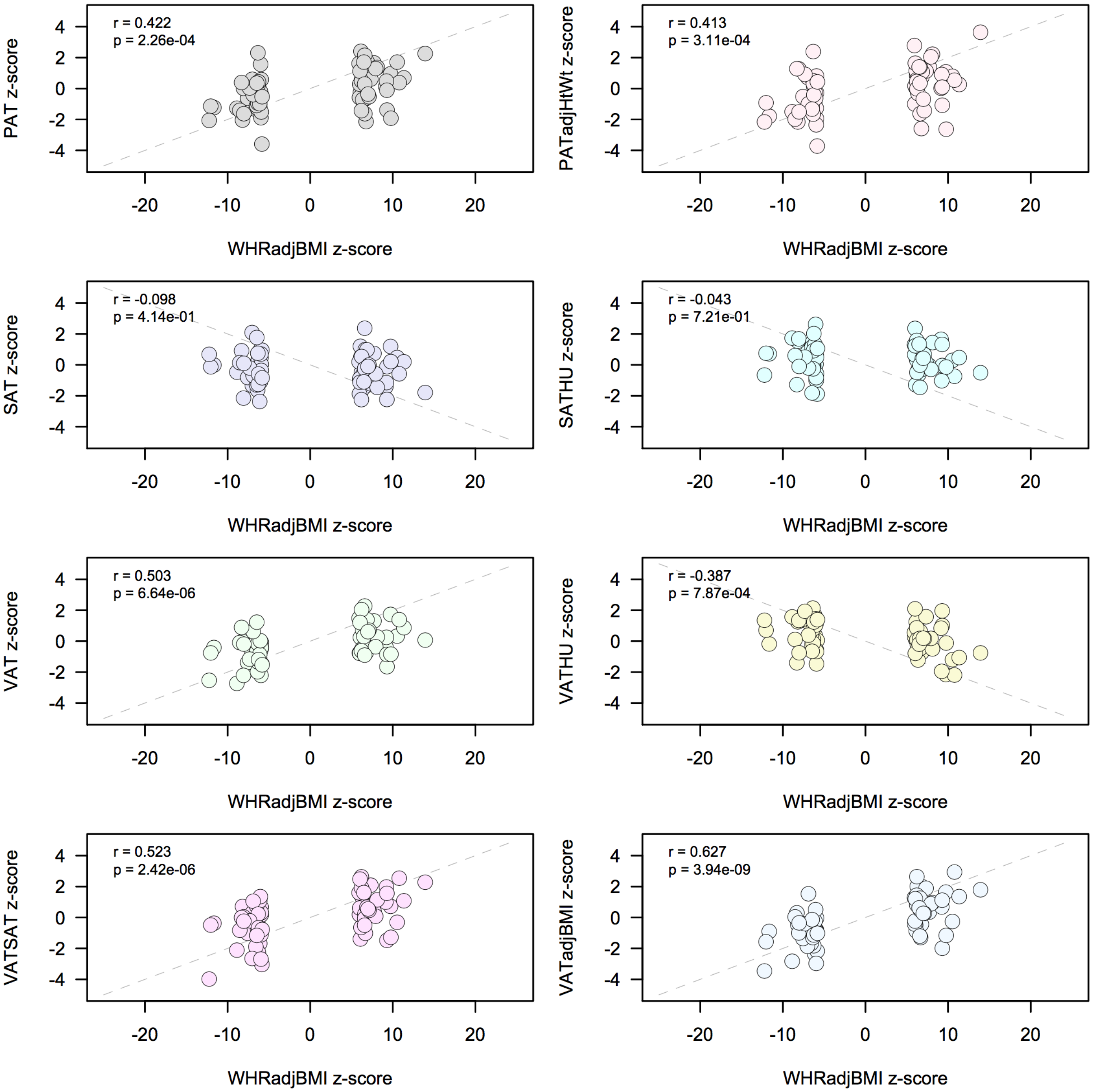

r = 0.422
p = 2.26e-04
PAT z-score
WHRadjBMI z-score
r = 0.413
p = 3.11e-04
PATadjHtWt z-score
WHRadjBMI z-score
r = -0.098
p = 4.14e-01
SAT z-score
WHRadjBMI z-score
r = -0.043
p = 7.21e-01
SATHU z-score
WHRadjBMI z-score
r = 0.503
p = 6.64e-06
VAT z-score
WHRadjBMI z-score
r = -0.387
p = 7.87e-04
VATHU z-score
WHRadjBMI z-score
r = 0.523
p = 2.42e-06
VATSAT z-score
WHRadjBMI z-score
r = 0.627
p = 3.94e-09
VATadjBMI z-score
WHRadjBMI z-score

Supplementary Figure 9 | Phenotypic distributions in UK Biobank sample. We extracted waist-to-hip (WHR) measures and body mass index (BMI) measures from the UK Biobank phenotype information, as well as a number of important phenotype-level covariates: age at assessment, sex, and UK Biobank assessment centre. We generated phenotypes in a manner consistent with previous efforts in the GIANT consortium [2,3,7]. We regressed each of the WHR and BMI phenotypes on age at assessment, age at assessment squared, assessment centre, and sex. To generate the WHR adjusted for BMI (WHRadjBMI) phenotype, we additionally included BMI as a covariate. We extracted the residuals from each of these regressions and then inverse normalized the residuals, to result in the final phenotype for analysis. Here, we show all phenotypes before and after standardisation. Phenotype conversions by sex were performed in sex-specific analysis groups.

a. Phenotype distributions in body mass index

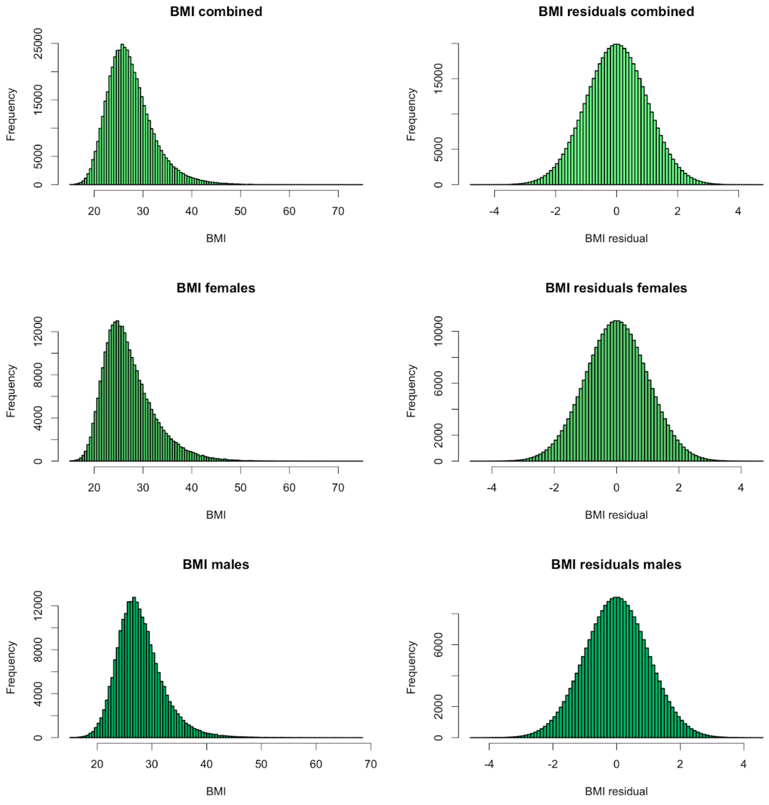

b. Phenotype distributions in waist-to-hip ratio, including adjustment for BMI

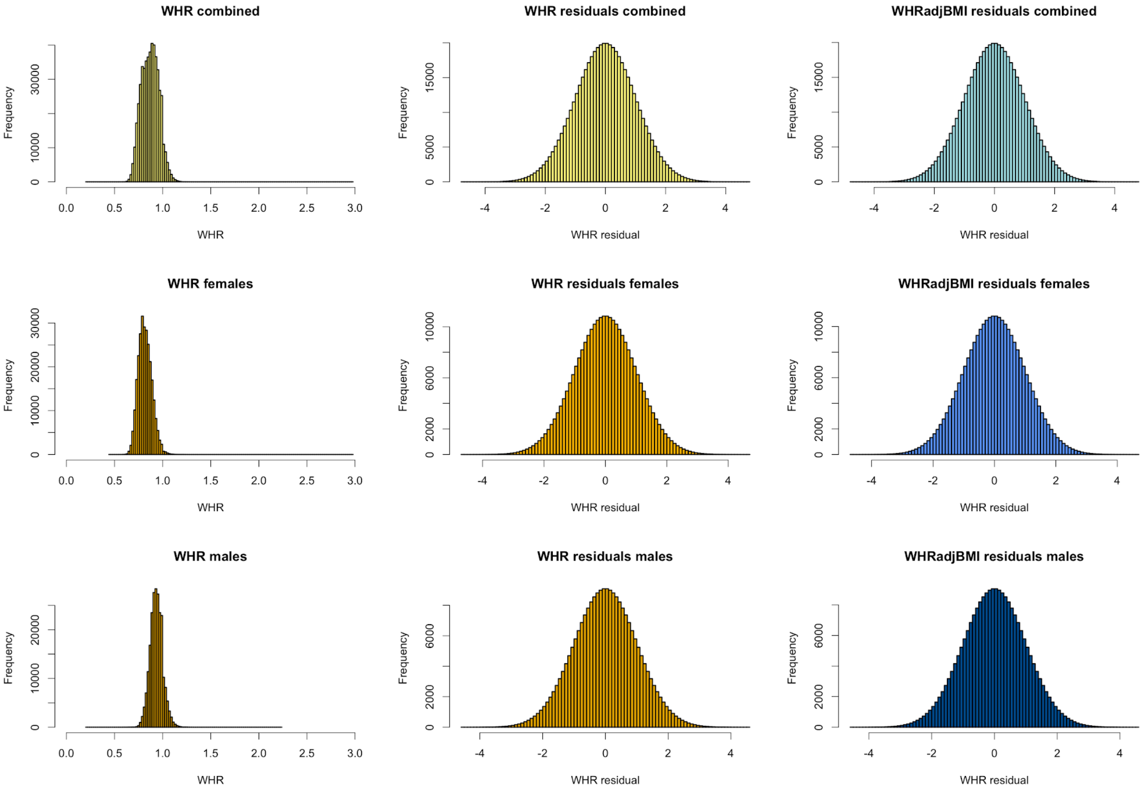

Supplementary Figure 10 | Correlation between various LD Score reference panels to use in BOLT-LMM. Before performing genome-wide association testing, we performed sensitivity testing in BOLT-LMM to optimize the data used in the genetic relationship matrix as well as the LD Score (LDSC) reference panel used (**Supplementary Methods**). We calculated correlation of SNP LD scores across all these panels to evaluate stability of the LD score metric and decided to use either the 'Baseline' panel [8,9] or custom UK Biobank panel for further sensitivity testing. Shading indicates Pearson's correlation (r) on a [-1,1] scale where darker blue shading indicates stronger positive correlation.

eur, LD scores calculated from European-ancestry samples in 1000 Genomes Phase 1; base, LD scores calculated in a 'baseline' model using 1000 Genomes Phase 3; genotyped, LD scores calculated with genotyped SNPs; imputed, LD scores calculated from imputed dosages converted to best-guess genotypes; 1k/5k/10k, the number of samples used to estimate LD Scores.

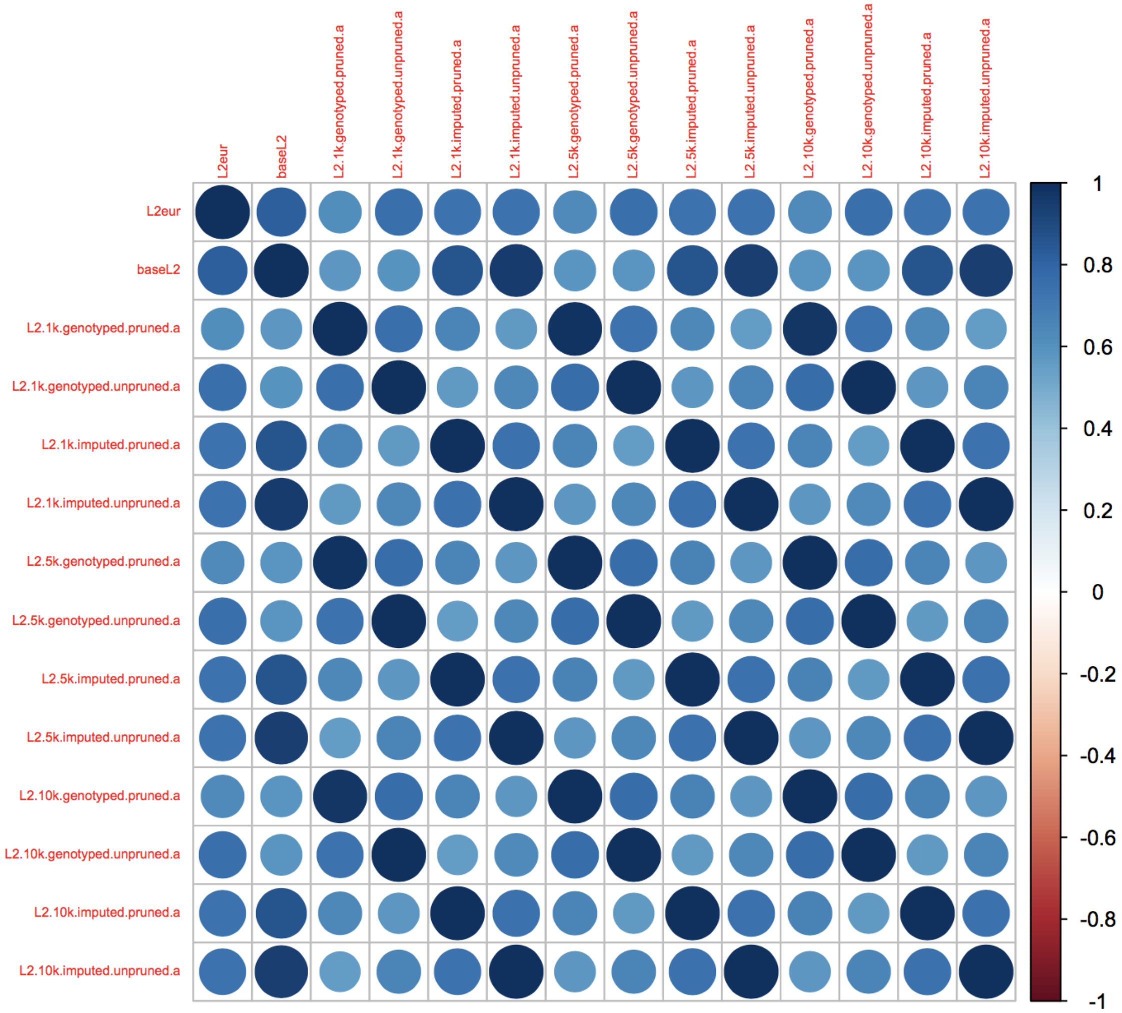

Supplementary Figure 11 | GWAS in obesity and fat distribution traits in UK Biobank only (combined sample). Manhattan plots for GWAS of body mass index (BMI), waist-to-hip ratio and waist-to-hip ratio adjusted for BMI in UK Biobank are shown. Note that y-axes are not continuous. Lines indicate traditional genome-wide significance ($p < 5 \times 10^{-8}$) and genome-wide significance in this analysis (blue line; $p < 5 \times 10^{-9}$).

a. Analysis of body mass index

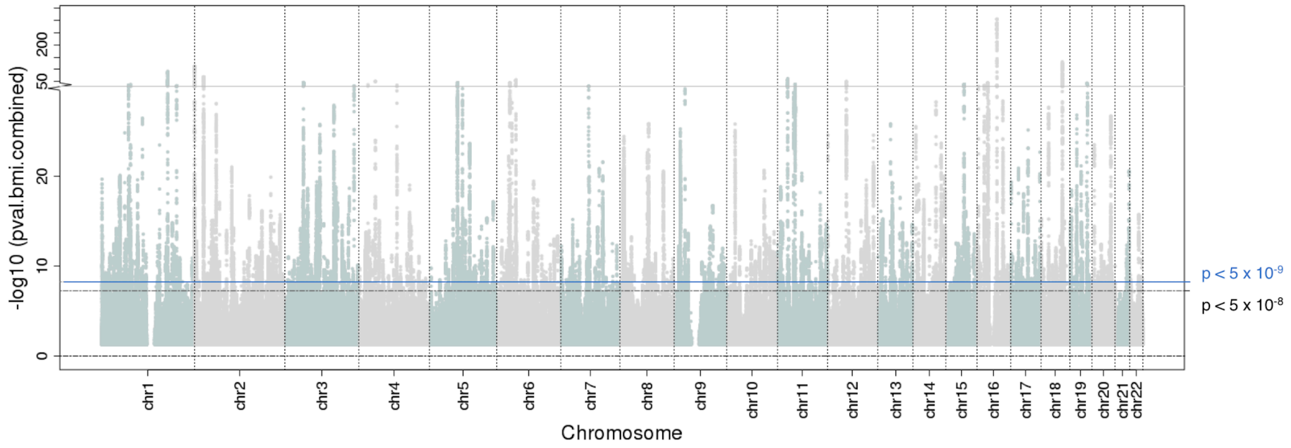

b. Analysis of waist-to-hip ratio

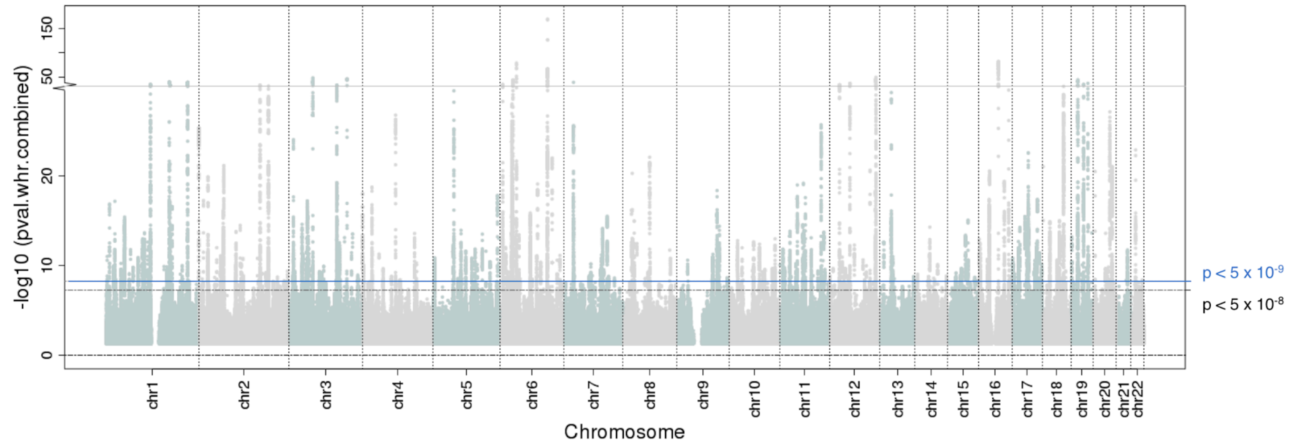

c. Analysis of waist-to-hip ratio adjusted for BMI

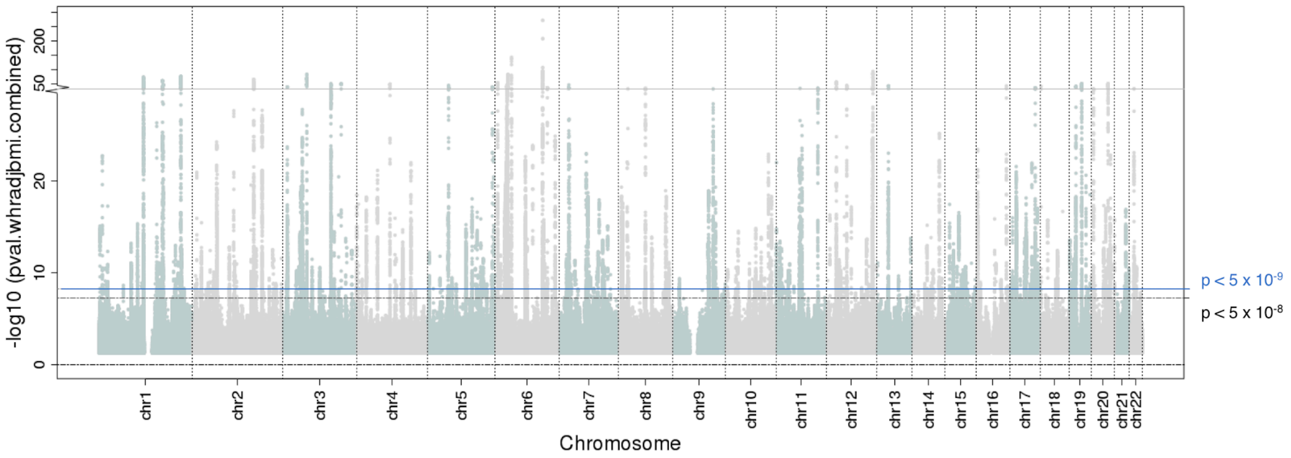

Supplementary Figure 12 | Genome-wide association testing in obesity and fat distribution traits in UK Biobank (sex-specific analyses). We performed genome-wide association testing in the UK Biobank samples for: body mass index (BMI), waist-to-hip ratio (WHR) and WHR adjusted for BMI (WHRadjBMI). The resulting Miami plots and quantile-quantile (QQ) plots show the results in the sex-specific analyses.

a. Analysis of body mass index

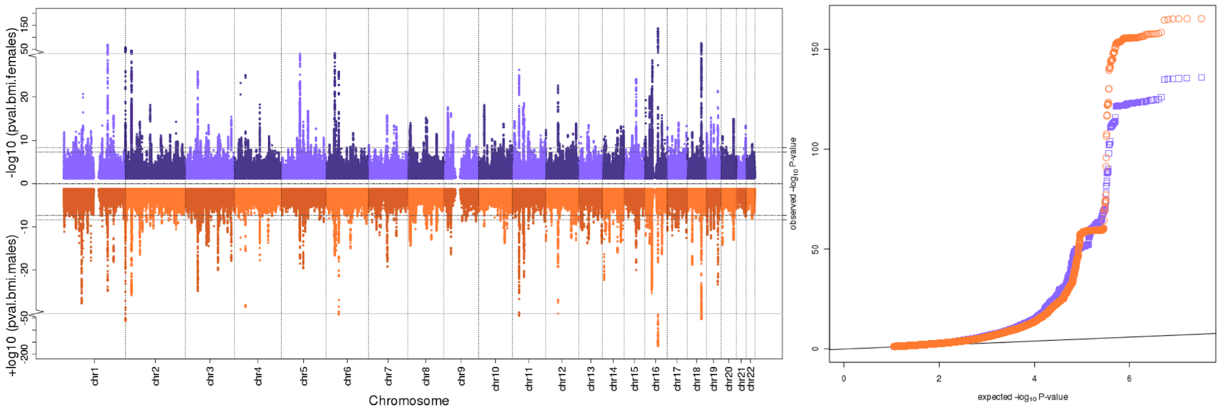

b. Analysis of waist-to-hip ratio

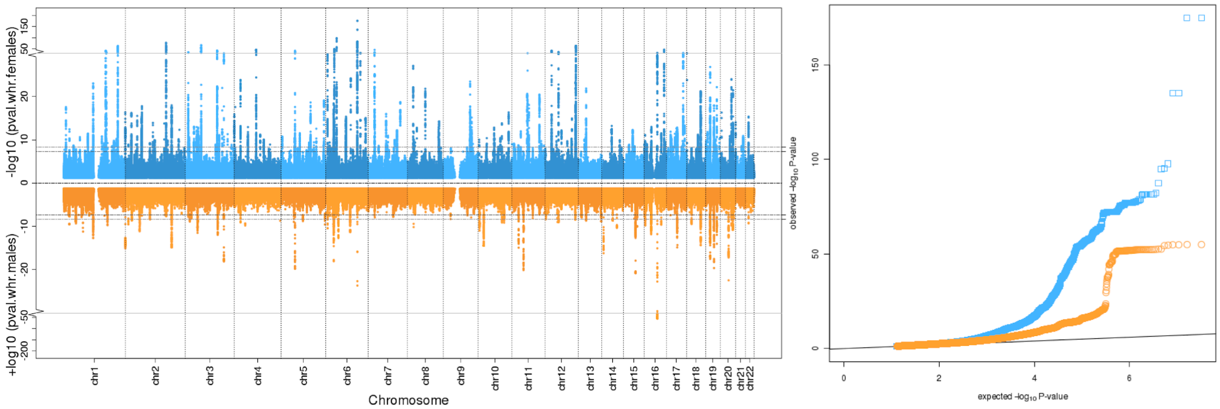

c. Analysis of waist-to-hip ratio adjusted for BMI

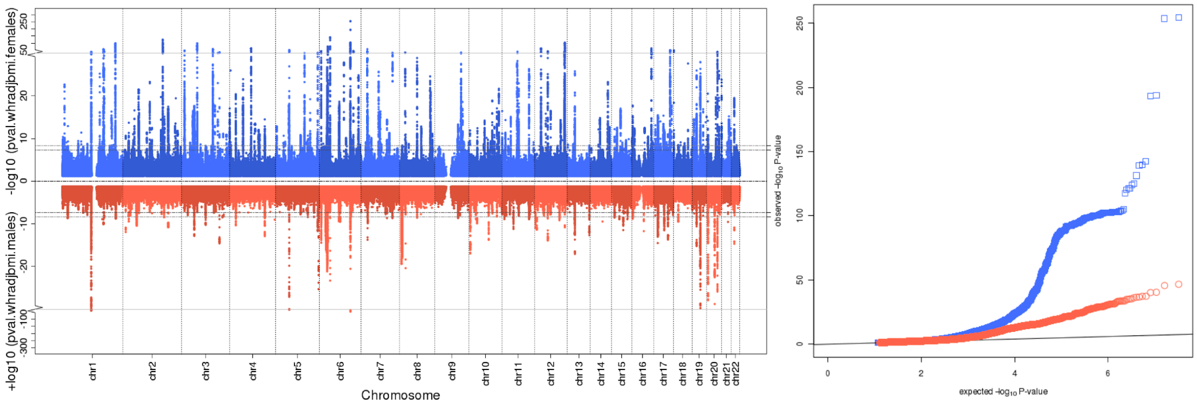

Supplementary Figure 13 | Concordance check of previously-implicated loci in genome-wide association studies in UK Biobank. After completing our GWAS in UK Biobank, we looked up the previously-described loci in BMI, WHR and WHRadjBMI (49 loci) and BMI (97 loci) from an effort by the GIANT consortium in 2014 [2,3]. We checked concordance between the previous associations and our data by examining minor allele frequency, effect size (beta), standard error, and $-\log_{10}$(p-value). Concordance checks and the correlation (Pearson's r) between UK Biobank and the previously-reported GIANT loci are shown for BMI, WHR and WHRadjBMI are shown.

a. Body mass index, previously-known loci in GIANT (x-axis) vs UK Biobank (y-axis)

Combined samples

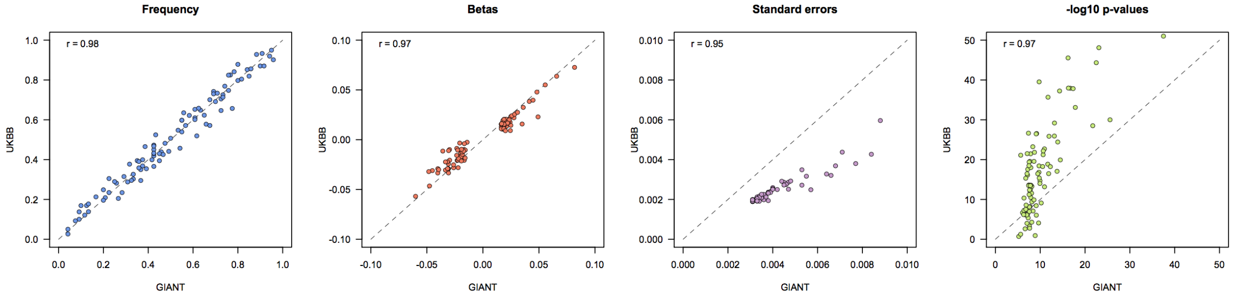

Women only

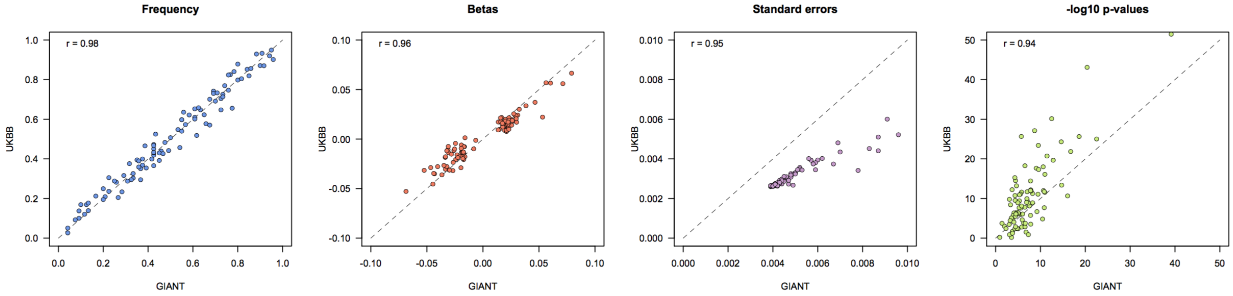

Men only

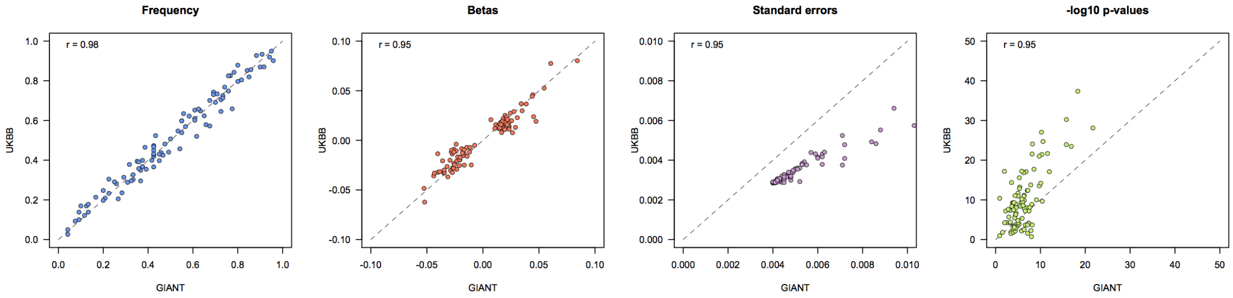

b. Waist-to-hip ratio, previously-known loci in GIANT (x-axis) vs UK Biobank (y-axis)

Combined samples

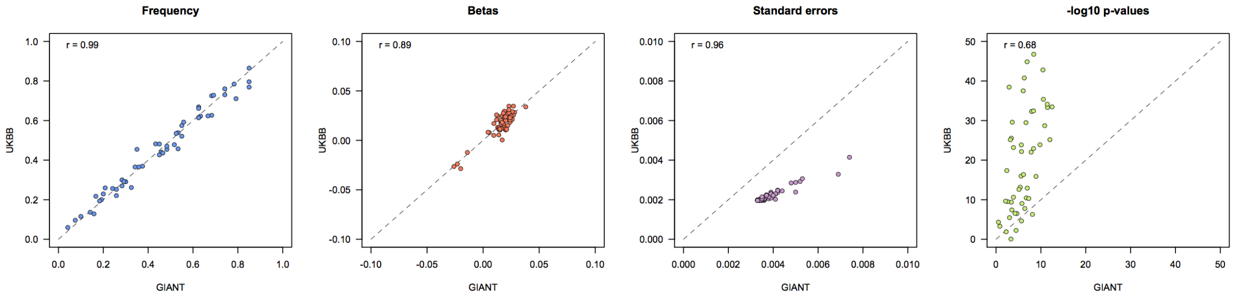

Women only

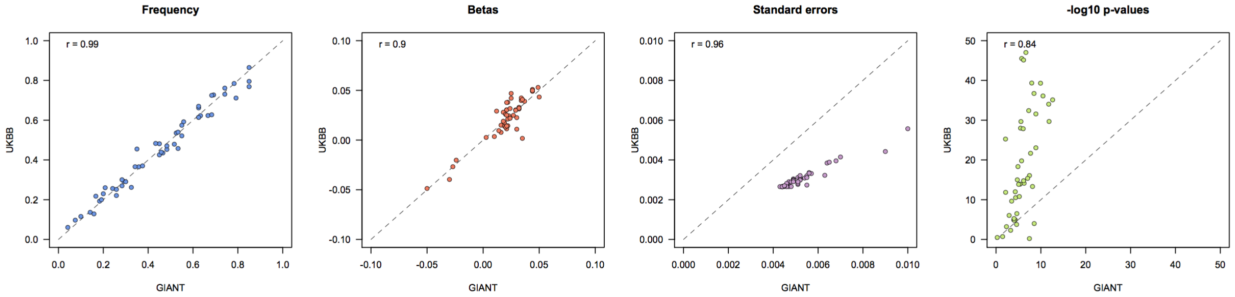

Men only

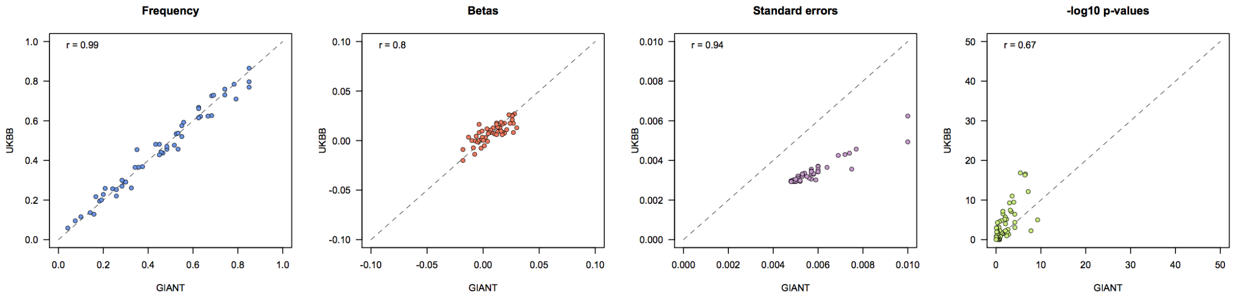

c. Waist-to-hip ratio adjusted for body mass index, previously-known loci in GIANT (x-axis) vs UK Biobank (y-axis)

Combined samples

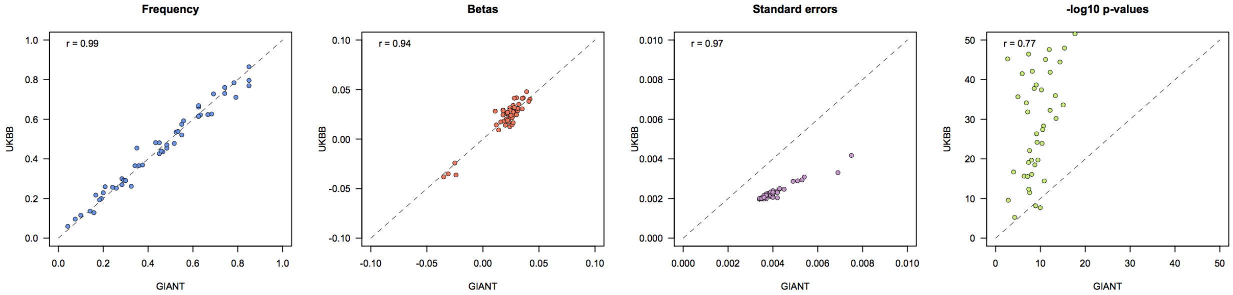

Women only

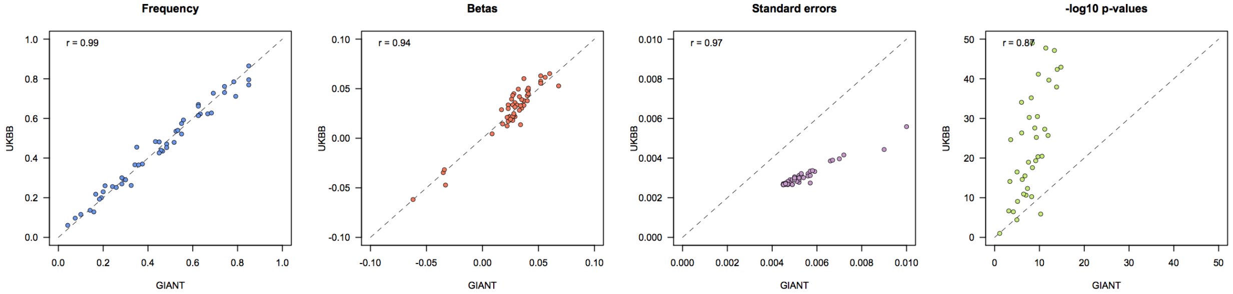

Men only

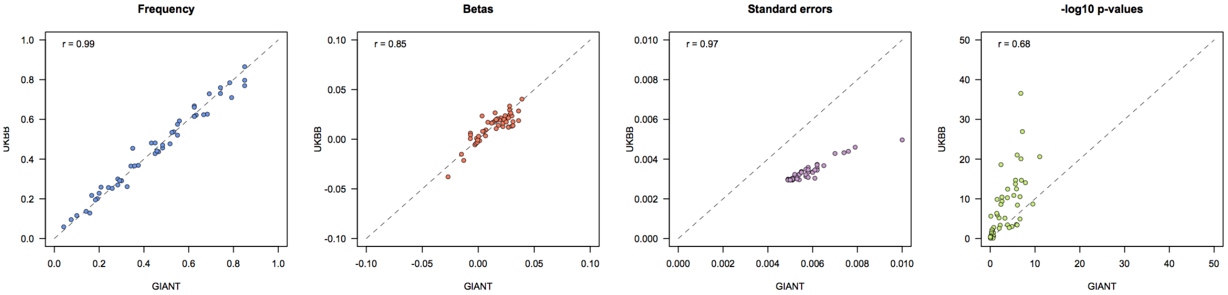

Supplementary Figure 14 | Allele frequency comparison in GIANT vs UK Biobank. Before performing meta-analyses of fat distribution and obesity phenotypes in GIANT and UK Biobank, we first compared SNP frequencies for all SNPs that appear in both studies (approximately all SNPs represented in the HapMap 2 resource [2,3,10,11]). Frequency comparisons (and downstream meta-analyses) were performed using the European-ancestry results generated by the GIANT consortium. All SNPs with a frequency difference > 15% between the two studies were dropped from all meta-analyses. Grey points: all SNPs represented in the comparison. Green SNPs: SNPs with a frequency difference > 10%. Blue points: SNPs with a frequency difference > 15%. Purple points: SNPs with a frequency difference > 20%.

BMI, body mass index; WHR, waist-to-hip ratio; WHRadjBMI, waist-to-hip ratio adjusted for BMI.

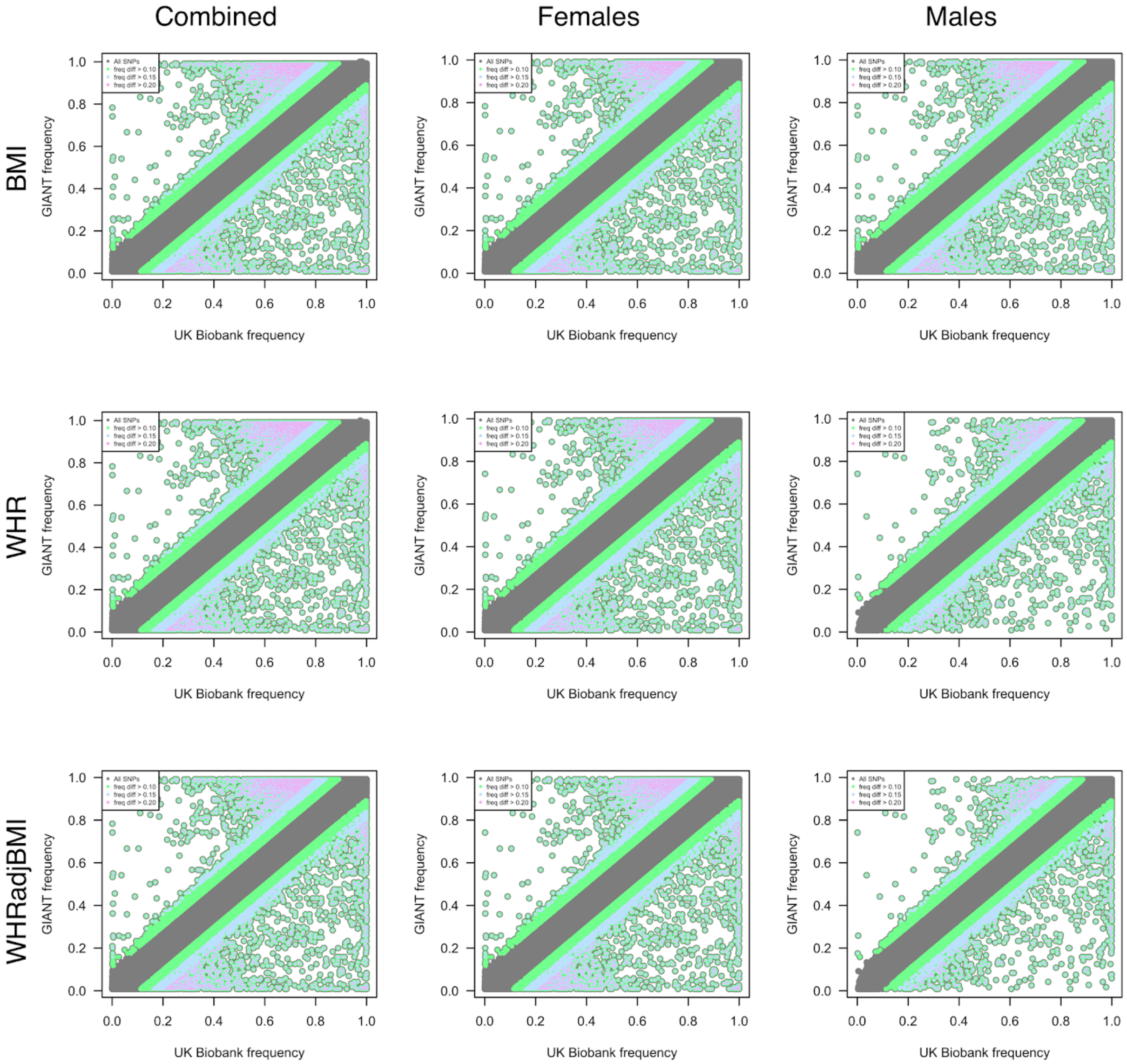

Supplementary Figure 15 | Phenotype correlations in UK Biobank. Correlations between a variety of fat distribution phenotypes collected in the UK Biobank.

WC, waist circumference; HC, hip circumference; BMI, body mass index; DXA, dual energy X-ray absorptiometry; res_WHRadjBMI_inv, inverse standardized residuals for waist-hip ratio adjusted for BMI (phenotype used in the WHRadjBMI GWAS).

a. In the combined sample

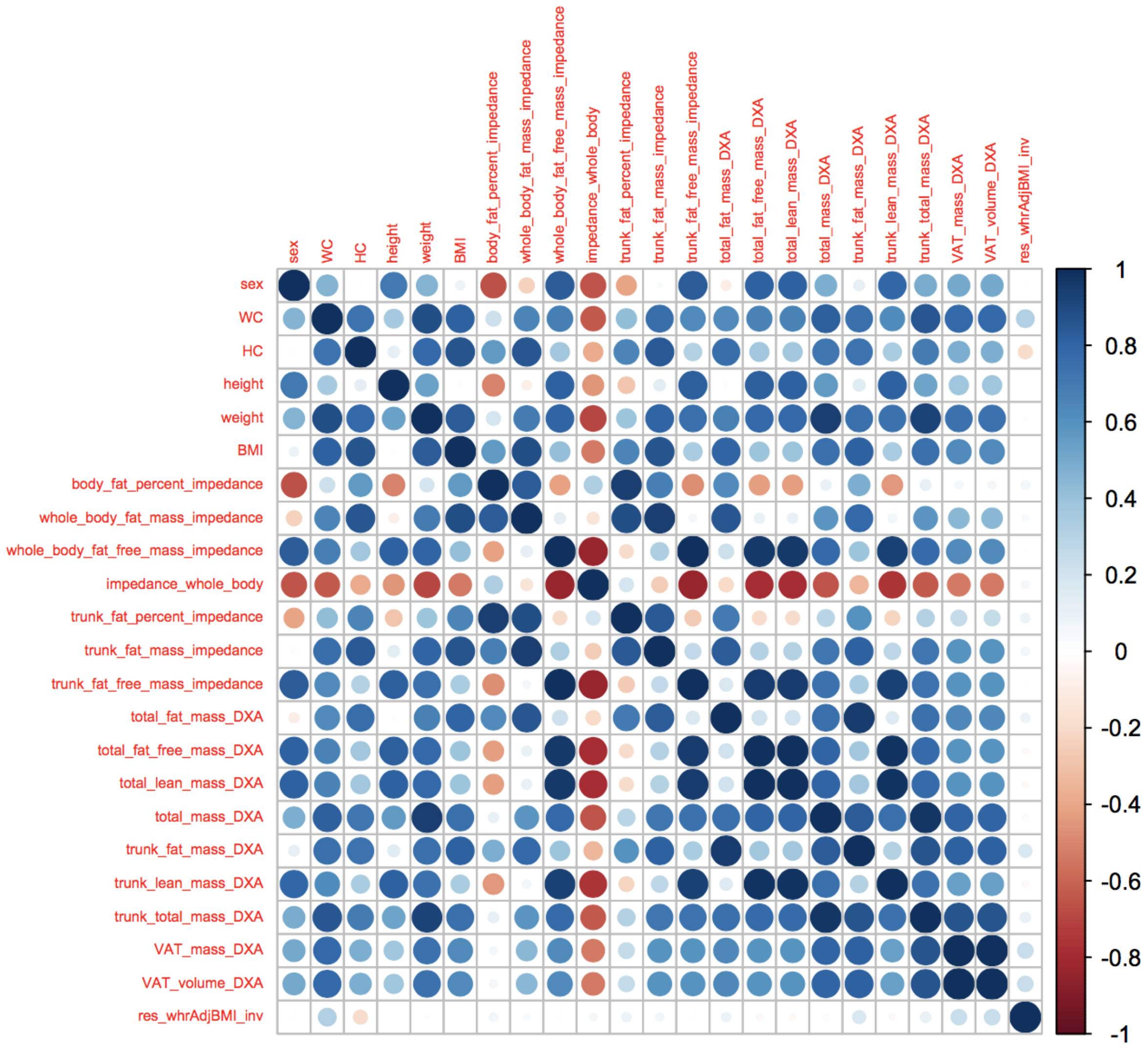

b. In females only

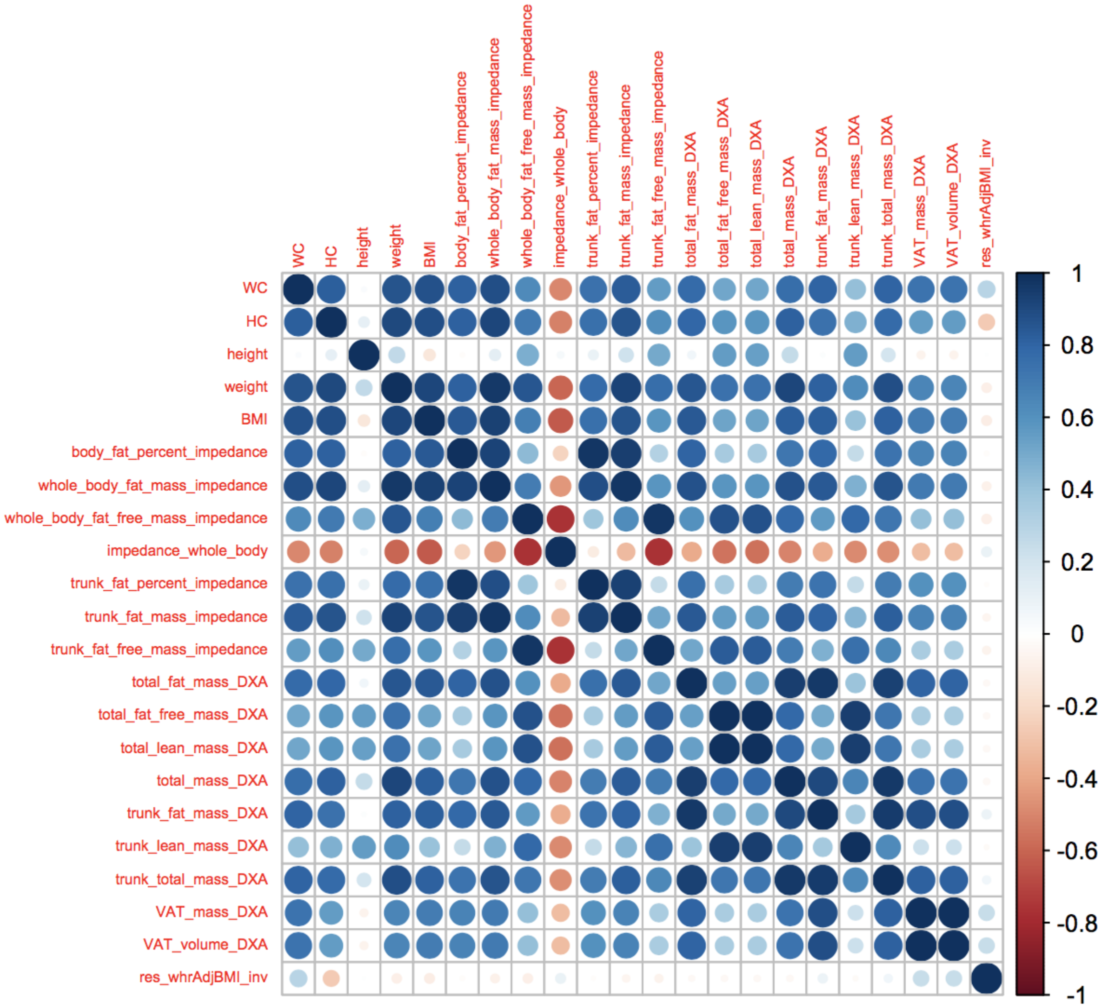

c. In males only

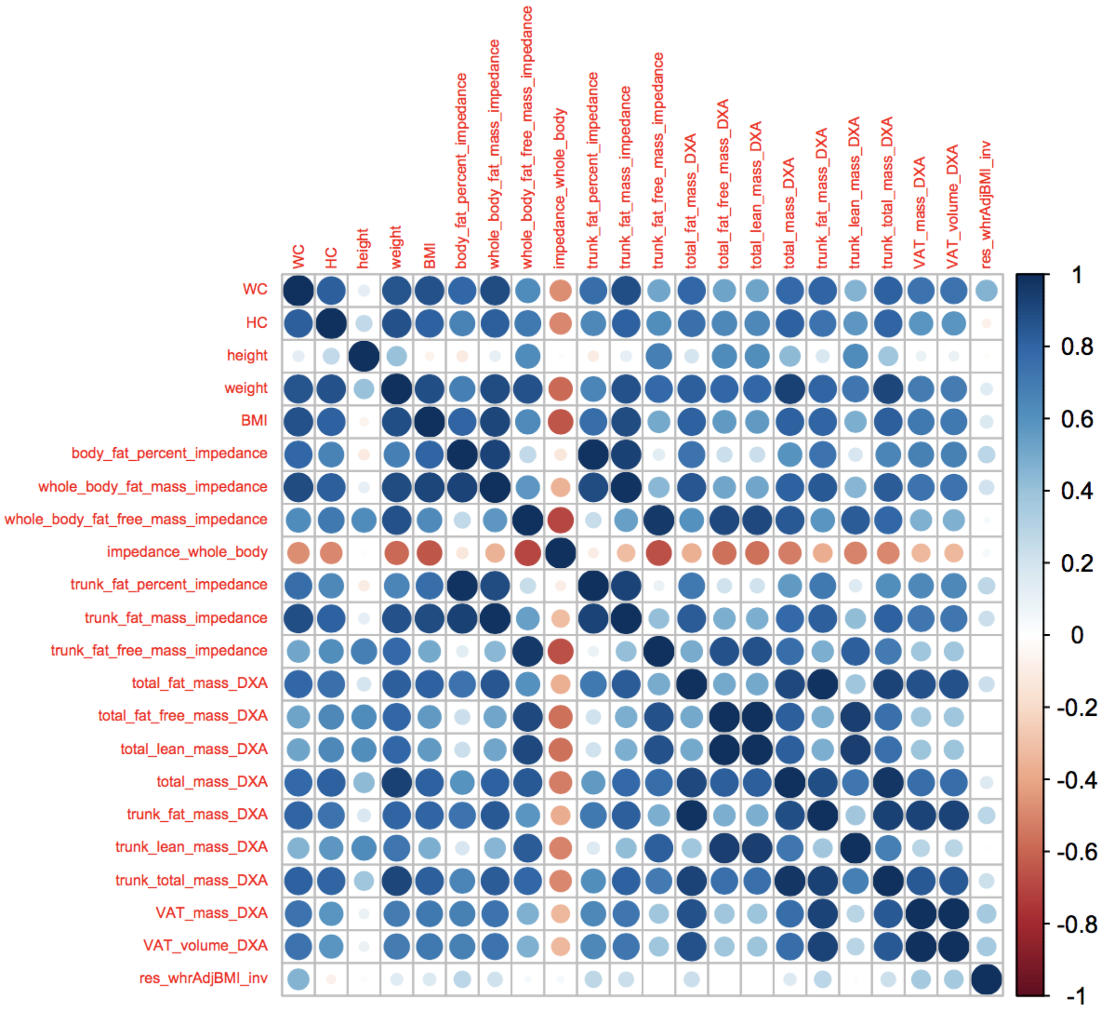

Supplementary Tables

Supplementary Table 1 | Summary-level statistics for index and secondary SNPs discovered in the combined and sex-specific meta-analyses. The tables containing summary-level data for associated SNPs are provided as downloadable text files from the project's GitHub repository (https://github.com/lindgrengroup/fatdistnGWAS). The file names are as follows:

(1) SuppTable1/whradjbmi.giant-ukbb.meta.1.merged.indexSnps.combined.parsed.txt
(2) SuppTable1/whradjbmi.giant-ukbb.meta.1.merged.secondarySnps.combined.parsed.txt

Files for the results from waist-to-hip ratio ('whr' in file name) are also provided. The 'indexSnps' file contains only the index SNPs for that trait. The 'secondarySnps' file contains all of the secondary SNPs for that trait (as determined using joint-conditional analysis in GCTA; see Methods in the main paper).

The column names in these files are:

SNP	dbSNP 151 marker name, following the rsID:alternate allele:reference allele scheme
Chr	Chromosome
Pos	Position, hg19 coordinates
A1	First (tested) allele. Not necessarily the alternate allele.
A2	Second allele. Not necessarily the reference allele
frqA1.combined	Frequency of A1 in the combined sample
beta.combined	Beta (for A1) in the combined analysis
se.combined	Standard error in the combined analysis
pval.combined	P-value from the meta-analysis, combined analysis
dir.combined	Directions of the betas in the meta-analysis. First column, UK Biobank. Second column, GIANT.
nmeta.combined	Meta-analysis sample size in the combined analysis
info.combined	The imputation score in the meta-analysis (taken from UK Biobank, as GIANT data does not include imputation quality).
Next 14 columns	Frequency, beta, se, pval, dir, nmeta and info repeated for the female-only and male-only analyses
psexdiff	P-value from the test of sex-dimorphism between males and females (see **Methods** of the main paper for details)

Supplementary Table 2 | Genomic inflation (lambda) and Linkage Disequilibrium Regression Score (LDSC) Intercepts in genome-wide association studies and meta-analysis. A standard quality control step after performing a genome-wide association study is to check the calibration of the resulting p-values. A standard metric used to evaluate whether the results are well-calibrated (i.e., primarily following the null distribution with some indication of polygenicity) is genomic inflation (lambda, λ) [12]. Lambda is expected to be ~1, under the assumption that only a percentage of SNPs will show true association to the trait. A second metric, the LDSC intercept [13], has been used in more recent GWAS; large sample sizes and tremendous polygenicity make it difficult to understand if a lambda that deviates substantially from 1 is indicative of polygenicity or confounding. As a quality control step on our data, we calculated both lambda and LDSC intercept in the UK Biobank GWAS and in the meta-analysis results. All LDSC intercepts and lambdas were calculated using LD Scores generated using UK Biobank (as described for BOLT-LMM sensitivity testing; see **Supplementary Methods**).

Phenotype	Sex	UK Biobank GWAS		Meta-analysis	
		Lambda (λ)	LDSC Intercept (se)	Lambda (λ)	LDSC Intercept (se)
BMI	Combined	1.4281	1.0395 (0.0057)	1.4034	1.0333 (0.0056)
	Women	1.2531	1.0289 (0.0045)	1.9095	1.0528 (0.0219)
	Men	1.2531	1.0219 (0.0043)	1.2564	1.0159 (0.0042)
WHR	Combined	1.3101	1.0336 (0.0051)	1.2966	1.0279 (0.005)
	Women	1.2005	1.0393 (0.0046)	1.207	1.0343 (0.0045)
	Men	1.1459	1.0207 (0.0036)	1.1747	1.0163 (0.0036)
WHRadjBMI	Combined	1.2531	1.0393 (0.0056)	1.2631	1.0346 (0.0054)
	Women	1.2005	1.0367 (0.0054)	1.2234	1.0334 (0.0052)
	Men	1.1459	1.018 (0.0038)	1.1587	1.0151 (0.0037)

Supplementary Table 3 | Test of collider bias at genome-wide associated SNPs. Conditioning a variable on a second, correlated variable (sometimes called conditioning on a 'collider') can induce both false-positive and false-negative associations [4,5]. Body mass index (BMI) and waist-to-hip ratio (WHR) correlate to one another (see **Supplementary Figures 4** and **15**); therefore, conditioning WHR on BMI to generate the waist-to-hip ratio adjusted for BMI (WHRadjBMI) phenotype may have resulted in collider bias at genome-wide associated SNPs. We examined the association statistics of WHRadjBMI index SNPs in meta-analyses of BMI and WHR (see **Supplementary Methods**). Here, we provide association statistics for WHRadjBMI-associated SNPs extracted from meta-analyses of BMI and WHR. The tables containing these data are provided as downloadable text files from the project's GitHub repository (https://github.com/lindgrengroup/fatdistnGWAS). The file names are as follows:

(1) SuppTable3/collider.bias.combined.index.results.txt
(2) SuppTable3/collider.bias.females.index.results.txt
(3) SuppTable3/collider.bias.males.index.results.txt

Each file contains the genome-wide significant index SNPs from the specific meta-analysis (combined samples, women only, or men only). Summary-statistics are always extracted from the same set of samples (e.g., all statistics in collider.bias.females.index.results.txt are extracted from the women-only analyses of WHRadjBMI, WHR and BMI).

The column names in these files are:

SNP	dbSNP 151 marker name, following the rsID:alternate allele:reference allele scheme
A1	The A1 allele, to which all statistics are aligned
frqA1.whradjbmi	Frequency of A1 in the whradjbmi analysis
beta.whradjbmi	Beta (for A1) in the whradjbmi analysis
se.whradjbmi	Standard error in the whradjbmi analysis
pval.whradjbmi	P-value from the whradjbmi meta-analysis
frqA1.bmi	Frequency of A1 in the bmi analysis
beta.bmi	Beta (for A1) in the bmi analysis
se.bmi	Standard error in the bmi analysis
pval.bmi	P-value from the bmi meta-analysis
frqA1.whr	Frequency of A1 in the whr analysis
beta.whr	Beta (for A1) in the whr analysis
se.whr	Standard error in the whr analysis
pval.whr	P-value from the whr meta-analysis

Supplementary Table 4 | Summary-statistics from the directional consistency analysis in EXTEND. The table containing the results from our directional consistency analysis for the 346 index SNPs are provided as a downloadable text file from the project's GitHub repository (https://github.com/lindgrengroup/fatdistnGWAS). The file names is as follows:

(1) SuppleTable4/Directional_consistency_346_whradjbmi_index_snps_GIANTUKBMA_EXTEND.txt

The association statistics in EXTEND were calculated by carrying out a linear regression model of WHRadjBMI on each SNP. All betas have been aligned to the WHRadjBMI increasing allele from our main meta-analysis. GIANTUKB_MA columns refer to data obtained from our main meta-analysis and EXTEND refers to estimates obtained from EXTEND dataset.

The column names in this file are:

RSID	dbSNP 151 marker name, following the rsID:alternate allele:reference allele scheme
Chr_pos	The chromosome and position of the SNP
A1_GIANTUKB_MA	A1 in the meta-analysis analysis
A2_GIANTUKB_MA	A2 in the meta-analysis analysis
FREQ_A1_GIANKUKB_MA	Frequency of A1 in the meta-analysis
BETA_GIANTUKB_MA	Beta in the meta-analysis (corresponding to A1)
SE_GIANTUKB_MA	Standard error of the beta
P_GIANTUKB_MA	P-value of the SNP
Direction_GIANTUKB_MA	Beta direction in GIANT and UK Biobank
N_GIANTUKB_MA	Total sample size
EXTEND_beta_A1	Beta for A1 in EXTEND
beta.whr	Beta (for A1) in the whr analysis
EXTEND_SE	Standard error of the beta in EXTEND
EXTEND_P	EXTEND p-value
Consistency	If the betas are consistent across the meta-analysis and EXTEND (TRUE/FALSE)

Supplementary Table 5 | Number and percentage of signals showing directional consistency in EXTEND dataset for all 346 index signals (46 known and the 300 novel). We tested for consisent direction of effect of the known and novel index SNPs from our meta-analysis in an independent sample set. We estimated consistency by calculating the effect of the WHRadjBMI-increasing alleles on WHRadjBMI in EXTEND (N = 7.721). Of the signals, 78% of the novel and 73.9% of the known signals showed directional consistency.

Signal type	Total N of signals	N betas directionally consistent	N betas directionally inconsistent	% showing directional consistency	% showing directional inconsistency
All	346	268	78	77.5%	22.5%
Novel	300	234	66	78.0%	22.0%
Known	46	34	12	73.9%	26.1%

Supplementary Table 6 | Heritability estimates across common SNPs and all SNPs in UK Biobank. We estimated heritability in waist-to-hip ratio (WHR), WHR adjusted for BMI (WHRadjBMI), and BMI using two different genomic relationship matrices (GRMs). The construction of these GRMs is described in the Methods and Supplementary Methods. In brief, we selected a high-quality set of SNPs to construct the GRM. For the first GRM to estimate heritability, we used the same as the GRM used in our association testing, using only SNPs with a minor allele frequency (MAF) > 1%. For the second, we used all SNPs, regardless of their frequency. We found heritability to be sex-dimorphic in WHR and WHRadjBMI but not in BMI. Including all SNPs rather than only common SNPs only minorly changed our heritability estimates. The test of sex-dimorphism is described in the Methods of the main paper.

Phenotype	Sex	N	h^2g (se)		Test for sex-dimorphism	
			GRM MAF > 1%	GRM all SNPs	GRM MAF > 1%	GRM all SNPs
WHRadjBMI	Combined	484,563	0.174 (0.002)	0.206 (0.002)	--	--
	Women	262,759	0.256 (0.003)	0.293 (0.004)	z = 19.51 p = 9.01×10^{-85}	z = 21.56 p = 3.95×10^{-103}
	Men	221,804	0.167 (0.003)	0.166 (0.004)		
WHR	Combined	485,486	0.194 (0.002)	0.227 (0.002)	--	--
	Women	263,148	0.254 (0.003)	0.277 (0.004)	z = 9.83 p = 8.12×10^{-23}	z = 10.68 p = 1.21×10^{-26}
	Men	222,338	0.208 (0.003)	0.213 (0.004)		
BMI	Combined	484,680	0.279 (0.002)	0.327 (0.002)	--	--
	Women	262,817	0.300 (0.003)	0.335 (0.004)	z = -1.175 p = 0.240	z = -1.823 p = 0.068
	Men	221,863	0.305 (0.004)	0.347 (0.005)		

Supplementary Table 7 | Summary statistics for the effect of WHRadjBMI index SNPs on BF% and WHR in UK Biobank individuals. The table contains summary-level data for the effect of 346 WHRadjBMI index SNPs on body fat percentage (BF%) and waist-to-hip ratio (WHR) in males and females combined provided as downloadable text files from the project's GitHub repository (https://github.com/lindgrengroup/fatdistnGWAS). The file names are as follows:

(1) SuppleTable7/WHRadjBMI_index_snps_with_BF_and_WHR_statistics.txt

Columns contain all association statistics for the effect of each index SNP on WHRadjBMI from the combined meta-analysis and BF% and WHR in combined analysis from a GWAS on 449,001 unrelated European-ancestry UK Biobank individuals. The columns are named as following [trait]_[analysis]_statistic e.g bf_combined_beta. All effects are given for WHR increasing alleles from the combined GWAS on UK Biobank individuals.

Supplementary Table 8 | Summary statistics for the effect of sex-dimorphic WHRadjBMI index SNPs on BF% in UK Biobank individuals. The table contains summary-level data for the effect of 105 sexually dimorphic SNPs on BF% in males and females separately provided as downloadable text files from the project's GitHub repository (https://github.com/lindgrengroup/fatdistnGWAS). The file name is as follows:

(1) SuppleTable8/WHRadjBMI_dimorphic_snps_merged_bfp_association_statistics.txt

The table contains columns with the association statistics for the effect of 105 dimorphic SNPs on WHRadjBMI and BF% in sex specific analysis. Columns are named with corresponding phenotype, sex and statistics given e.g bf_male_beta. All effects are given for WHRadjBMI increasing alleles from the combined meta-analysis results. The column named Male_or_female_specific indicates whether the SNP is having a greater effect in females compared to males based on the criteria described in the '*Identification of sex-dimorphic signals*' section of the **Methods** section of the main paper. Male-specific SNPs are denoted MALE and female-specific SNPs denoted FEMALE.

Supplementary Table 9 | Number of WHRadjBMI index SNPs showing strong associations with BF% and the direction of effect. Association statistics between WHRadjBMI index SNPs and BF% were obtained from UK Biobank individuals. Amongst the 346 WHRadjBMI index SNPs from the combined meta-analysis, 59 SNPs were strongly associated with BF% in the combined GWAS (based on a Bonferroni corrected p-value of $0.05/346 = 1.44 \times 10^{-4}$); 34 of these were associated with increased BF% and 25 with decreased BF%. Of the 105 sex-dimorphic SNPs, 36 were strongly associated with BF% in the combined GWAS with 21 being associated with a higher BF% (based on a Bonferroni corrected p-value $0.05/105 = 4.8 \times 10^{-4}$).

SNPs		N associated With BF%	N associated with increased BF%	N associated with decreased BF%
		Bonferroni corrected $p < 1.44 \times 10^{-4}$		
346 index SNPs	Combined	59	34	25
	Female	36	22	14
	Male	44	25	19
		Bonferroni corrected $p < 4.8 \times 10^{-4}$		
105 dimorphic SNPs	Combined	36	21	15
	Female	26	17	9
	Male	30	17	13

Supplementary Table 10 | Summary of phenotypic information in the UK Biobank and GIANT samples. We used the UK Biobank resource to perform genome-wide association studies in obesity and fat distribution phenotypes. Summary information on the phenotypes are provided below. Additional information from the GIANT data, where available, is also provided. Note that the listed samples indicate the maximum number of samples available for testing; many of SNPs appear only in UK Biobank and not GIANT, and therefore can only be tested in the UK Biobank samples. Sample sizes reflect the total sample available for analysis (i.e., after sample exclusions were applied).

UKBB, UK Biobank; BMI, body mass index; WHR, waist-to-hip ratio; WHRadjBMI, waist-to-hip ratio adjusted for BMI.

Phenotype	Sex	UKBB N	GIANT N	Total N	Summary statistics from UK Biobank samples		
					Mean	Median	Standard deviation
WHR	Combined	485,486	212,248	697,734	0.871	0.873	0.090
	Women	263,148	118,004	381,152	0.817	0.813	0.070
	Men	222,338	94,434	316,772	0.935	0.933	0.065
WHRadjBMI	Combined	484,563	210,086	694,649	-	-	-
	Women	262,759	116,742	379,501	-	-	-
	Men	221,804	93,480	315,284	-	-	-
BMI	Combined	484,680	322,154	806,834	27.409	26.719	4.807
	Women	262,817	171,977	434,794	27.065	26.093	5.205
	Men	221,863	152,893	374,756	27.818	27.290	4.249

Supplementary Table 11 | Samples excluded from genome-wide association testing in UK Biobank. For genome-wide association testing in UK Biobank, we excluded: samples with withdrawn consent, the heterozygosity and missingness outliers (as identified by UK Biobank upon data release), samples with phenotypic vs genotypic sex mismatches, and samples with genotyped but not imputed data. We additionally performed a series of sensitivity genome-wide association studies, dropping (1) all related samples and (2) all related samples and all samples that were not white British individuals. Sample counts (potentially overlapping) and the exclusion criteria are provided below.

Exclusion criterion	Samples dropped
Consent withdrawn	18
Heterozygosity and missingness outliers	968
Phenotypic vs genotypic sex mismatch	378
Samples genotyped but not imputed	968
Not a white British sample	78,560
Related samples (kinship > 0.0442 (i.e., 3rd degree relative or higher)	107,162

Supplementary Table 12 | Configurations for sensitivity testing in BOLT-LMM. We performed a series of genome-wide association studies (GWAS) in the waist-to-hip ratio adjusted for body mass index (WHRadjBMI) phenotype. We varied either (a) the genetic relationship matrix (GRM) or (b) the LD Score reference panel (LDSC), to see which model yielded the best calibrated LD Score intercept and heritability estimate (h^2g) consistent with previous information. If the model is well-calibrated (i.e., the association statistics are well behaved), the LD Score intercept should be ~1. An LD Score intercept much larger than one is indicative of stratification due to relatedness, ancestral heterogeneity, or other sources. Where relevant, statistics are reported for both the 'infinitesimal' and 'non-infinitesimal' models in BOLT-LMM. The combination of a GRM calculated with imputed, pruned SNPs ($r^2 < 0.2$) and an LD Score reference panel calculated from UK Biobank (UKBB) yielded the heritability estimate closest to the current estimate, and yielded the best-calibrated LD Score intercept (highlighted in green).

GRM, genetic relationship matrix; LDSC, LD Score Intercept; non-inf model, non-infinitesimal model; inf model, infinitesimal model; int, intercept; λ, lambda (genomic inflation); 1KG EUR, 1000 Genomes (Phase I) European-ancestry samples.

GRM	LDSC panel	h^2g	Linear mixed model			
			LDSC int, inf model	LDSC int, non-inf model	λ inf model	λ non-inf model
Imputed SNPs, pruned	Baseline	0.173 (0.001)	1.253 (0.058)	1.248 (0.059)	1.32	1.32
Imputed SNPs, pruned	1KG EUR	0.138 (0.001)	1.143 (0.050)	1.137 (0.050)	1.35	1.35
Imputed SNPs, pruned	UKBB	0.173 (0.001)	1.031 (0.023)	1.0230 (0.023)	1.32	1.33
Imputed SNPs, unpruned	UKBB	0.162 (0.001)	1.144 (0.021)	1.136 (0.021)	1.61	1.61
Genotyped SNPs, pruned	UKBB	0.138 (0.001)	-0.329 (0.101)	-0.341 (0.100)	1.35	1.291
Genotyped SNPs, unpruned	UKBB	0.170 (0.001)	0.656 (0.035)	0.646 (0.035)	1.46	1.48

Supplementary Table 13 | Summary of samples analyzed for sensitivity testing GWAS in UK Biobank. To ensure that our initial analyses in UK Biobank were not confounded by relatedness or ancestral heterogeneity (and to check that the linear mixed model was properly accounting for this structure), we additionally ran GWAS in UK Biobank using: (1) only the unrelated samples, and (2) only the unrelated white British samples. We then meta-analyzed this data with the pre-existing data, to check the consistency of our index and secondary signals in these analyses. Sample sizes for these analyses are provided here.

UKBB, UK Biobank; BMI, body mass index; WHR, waist-to-hip ratio; WHRadjBMI, waist-to-hip ratio adjusted for BMI.

Phenotype	Sex	UKBB All (N)	UKBB unrelated (N)	UKBB white British unrelated (N)	GIANT N
WHR	Combined	485,486	403,838	334,454	224,459
	Women	263,148	218,085	179,857	118,004
	Men	222,338	185,753	154,597	94,434
WHRadjBMI	Combined	484,563	403,059	333,938	224,459
	Women	262,759	217,750	179,613	116,742
	Men	221,804	185,309	154,325	93,480
BMI	Combined	484,680	403,150	334,014	322,154
	Women	262,817	217,795	179,648	171,977
	Men	221,863	185,355	154,366	152,893

Supplementary Table 14 | Summary characteristics of EXTEND dataset. The EXTEND (Exeter 10,000) sample collection contains genetic and phenotypic data for 7,721 individuals of white European descent from South West England. Summary information has been calculated on all individuals with available phenotypic data (N = 7,537).

Waist-to-hip ratio, WHR; body mass index, BMI; T2D, type 2 diabetes; T1D, type 1 diabetes.

Sex	N	Mean Age (min, max)	WHR (min, max)	Height (min, max)	BMI (min, max)	N of TD2 cases	N of T1D cases
Combined	7,537	56 (18, 98)	0.87 (0.58, 1.72)	1.67 (1.22, 2.02)	27.3 (14.27, 63.37)	1,424	203
Males	3,140	58 (18, 98)	0.94 (0.67, 1.28)	1.76 (1.41, 2.02)	27.89 (14.27, 62.11)	844	115
Females	4,397	55 (18, 94)	0.82 (0.58, 1.72)	1.63 (1.22, 1.90)	26.87 (14.44, 63.37)	580	88

Supplementary Table 15 | Summary of samples analyzed in the sensitivity meta-analysis of GIANT population-based studies only and UK Biobank. To ensure that no bias was introduced in our GIANT and UK Biobank meta-analysis by the inclusion of cases and controls in the original GIANT meta-analysis [3], we carried out a meta-analysis of GIANT population-based only studies and UK Biobank. A visualisation of this sensitivity analysis is shown in **Supplementary Figure 3** and a link to the summary-statistics for each study is provided in the 'Code and Data Release' section.

Phenotype	Sex	UKBB unrelated Europeans (N)	GIANT population-based studies only (N)	Total N
WHR	Combined	449,216	73,980	523,196
	Women	243,743	40,363	284,106
	Men	205,474	33,617	239,091
WHRadjBMI	Combined	449,216	73,925	523,141
	Women	243,743	40,334	284,077
	Men	205,474	33,266	238,740

Code and data release

Code

Relevant code for this project can be found in the following GitHub repository: https://github.com/lindgrengroup/fatdistnGWAS

This repository additionally includes Supplemental Tables 1, 2, 4, 8 and 9 provided in .txt format.

Summary-level data:

Note: the individual-level UK Biobank data (including imputed genotypes and phenotypes) are publicly-available upon submission of an application (http://www.ukbiobank.ac.uk/) and are therefore not released with this manuscript.

UK Biobank summary-level data

Downloadable summary-level data from the meta-analyses performed in this work (UK Biobank (UKBB) + GIANT) can be found here (and is in the process of being uploaded to the GIANT website, see *GIANT summary-level data* on the next page):

https://doi.org/10.5281/zenodo.1251813

This link is also provided at the project's GitHub repository (please see the README; https://github.com/lindgrengroup/fatdistnGWAS)

GIANT summary-level data

The summary-level data from previous meta-analyses performed by the GIANT consortium that we used in our meta-analyses can be downloaded here:

http://portals.broadinstitute.org/collaboration/giant/index.php/GIANT_consortium_data_files

See next page for specific links to each file.

Specifically, the files downloaded were:

GIANT meta-analysis	Download link
WHRadjBMI, combined sample	http://portals.broadinstitute.org/collaboration/giant/images/e/eb/GIANT_2015_WHRadjBMI_COMBINED_EUR.txt.gz
WHRadjBMI, women only	http://portals.broadinstitute.org/collaboration/giant/images/f/f8/GIANT_2015_WHRadjBMI_FEMALES_EUR.txt.gz
WHRadjBMI, men only	http://portals.broadinstitute.org/collaboration/giant/images/1/14/GIANT_2015_WHRadjBMI_MALES_EUR.txt.gz
WHR, combined sample	http://portals.broadinstitute.org/collaboration/giant/images/5/54/GIANT_2015_WHR_COMBINED_EUR.txt.gz
WHR, women only	http://portals.broadinstitute.org/collaboration/giant/images/d/de/GIANT_2015_WHR_FEMALES_EUR.txt.gz
WHR, men only	http://portals.broadinstitute.org/collaboration/giant/images/f/fd/GIANT_2015_WHR_MALES_EUR.txt.gz
BMI, combined sample	http://portals.broadinstitute.org/collaboration/giant/images/1/15/SNP_gwas_mc_merge_nogc.tbl.uniq.gz
BMI, women only	http://portals.broadinstitute.org/collaboration/giant/images/0/0d/Women_SNP_gwas_mc_merge_nogc.tbl.uniq.gz
BMI, men only	http://portals.broadinstitute.org/collaboration/giant/images/7/7b/Men_SNP_gwas_mc_merge_nogc.tbl.uniq.gz

Summary-level data from a genome-wide association study of ectopic fat depots

We looked up SNPs associated to WHRadjBMI in a recently-performed genome-wide association study (GWAS) of ectopic fat depots in a multi-ancestry sample [6]. GWAS was performed in 8 specific depots, and the data and links to that data are here:

https://grasp.nhlbi.nih.gov/FullResults.aspx

Chu et al meta-analysis	Download link
Pericardial adipose tissue (PAT)	https://grasp.nhlbi.nih.gov/downloads/FullResults/2017/2017_Chu/PAT.tar.gz
PAT adjusted for height and weight	https://grasp.nhlbi.nih.gov/downloads/FullResults/2017/2017_Chu/PATadjHtWt.tar.gz
Subcutaneous adipose tissue (SAT)	https://grasp.nhlbi.nih.gov/downloads/FullResults/2017/2017_Chu/SAT.tar.gz
SAT attenuation	https://grasp.nhlbi.nih.gov/downloads/FullResults/2017/2017_Chu/SATHU.tar.gz
Visceral adipose tissue (VAT)	https://grasp.nhlbi.nih.gov/downloads/FullResults/2017/2017_Chu/VAT.tar.gz
VAT adjusted for BMI	https://grasp.nhlbi.nih.gov/downloads/FullResults/2017/2017_Chu/VATadjBMI.tar.gz
VAT attenuation	https://grasp.nhlbi.nih.gov/downloads/FullResults/2017/2017_Chu/VATHU.tar.gz
Ratio of SAT and VAT	https://grasp.nhlbi.nih.gov/downloads/FullResults/2017/2017_Chu/VATSAT.tar.gz
Ratio of SAT and VAT adjusted for BMI	https://grasp.nhlbi.nih.gov/downloads/FullResults/2017/2017_Chu/VATSATadjBMI.tar.gz

Supplementary Methods and Results

Sensitivity testing in BOLT-LMM

Data for the genetic relationship matrix and LD Score reference panel

In implementing a linear mixed model, BOLT-LMM [14] requires three primary components: (1) the (imputed) genotype and phenotype data of the samples you wish to test; (2) a genetic relationship matrix (GRM), to estimate structure in the data due to relatedness, ancestral heterogeneity, or other factors; and (3) a reference panel of linkage disequilibrium scores (LDSC)[13], used to calibrate test statistics. Before beginning our genome-wide association studies (GWAS) in UK Biobank, we performed sensitivity testing in BOLT-LMM to ascertain which data should be used to populate the GRM, and which data to use as the LDSC reference panel.

We constructed four different GRMs: (1) genotyped data, unpruned SNPs; (2) genotyped data, SNPs pruned at $r^2 = 0.2$; (3) imputed data, unpruned SNPs; (4) imputed data, SNPs pruned at $r^2 = 0.2$. Imputed dosages in their bgen2 format (as distributed by UK Biobank) were converted to best-guess genotypes using Plink 1.9 [15,16], setting the hardcall threshold at 0.25. GRMs were always calculated using SNPs with:

- minor allele frequency (MAF) > 1%,
- imputation info score > 0.8 (for imputed SNPs only),
- missingness < 1%,
- Hardy-Weinberg $p > 1 \times 10^{-8}$,
- excluding the lactase locus on chromosome 2, the major histocompatibility complex (MHC) on chromosome 6, and inversions on chromosomes 8 and 17.

The coordinates for these regions are:

Chromosome	Name	Start (hg19)	Stop (hg19)
2	Lactase locus	129,883,530	140,283,530
6	MHC	24,092,021	38,892,022
8	inversion	6,612,592	13,455,629
17	inversion	40,546,474	44,644,684

We additionally tested three different LDSC reference panels. The first panel was derived from the European-ancestry 1000 Genomes [9,17] samples and is distributed with the LDSC software (https://github.com/bulik/ldsc). The second panel was called the 'baseline' LDSC reference, generated in work by Finucane et al [8] and computed using data from 1000 Genomes Phase 3 [9]. We constructed the third LDSC panel by calculating LD scores from best-guess genotypes in the UK Biobank data. To test the stability of these scores, we:

(1) Selected three sample sizes in which to calculate LD scores: 974 samples (0.1% of the UK Biobank data), 4,874 samples (1% of the UK Biobank data), and 9,748 samples (2% of the UK Biobank data)

(2) Randomly selected five sets of unrelated samples for each of these sample sizes (e.g., five different sets of 1,000 unrelated samples in UK Biobank)

(3) Calculated LD scores within each random set

(4) Calculated the correlation of the LD scores for these sample sets. LD Scores were calculated for either
    (a) Genotyped SNPs
    (b) Imputed SNPs

We found the LD scores to be highly stable across different sets of samples and SNPs (genotyped or imputed) in UK Biobank (**Supplementary Figure 10**), and therefore selected a panel calculated in 9,748 samples constructed either from genotyped SNPs or imputed SNPs converted to best-guess genotypes, as well as the 'baseline' panel to use in sensitivity testing.

Genome-wide association studies (GWAS) for sensitivity testing

We then ran a series of GWAS for sensitivity testing, altering the GRM and LDSC reference panel, to see which configuration seemed optimal given the data (**Supplementary Table 12**).

The combination of:

(1) A genetic relationship matrix calculated from imputed SNPs converted to best-guess genotypes and pruning SNPs at $r^2 = 0.2$, and
(2) An LDSC reference panel calculated from imputed SNPs converted to best-guess genotypes and pruning SNPs at $r^2 = 0.2$ in 9,748 UK Biobank samples

yielded the best-calibrated LD Score intercept (1.031) as well as a heritability estimate (17.3%) most consistent with current estimates for WHRadjBMI.

We therefore decided to run all of our GWAS in UK Biobank for BMI, WHR, and WHRadjBMI using this selection for GRM and LDSC reference panel.

Constructing an LD reference panel for locus identification and conditional testing

To identify top (i.e., index) signals and any secondary signals, we first performed linkage disequilibrium (LD)-based clumping [15,16], followed by conditional and joint analysis using GCTA [18].

LD Clumping

LD clumping (in Plink [15,16]) relies on (a) summary-level data from a genome-wide association study or meta-analysis and (b) a reference panel from which LD calculations can be performed. Calculating LD in the full UK Biobank (N ~ 500,000) is computationally expensive; therefore, we created a 'reference' set of data from the UK Biobank data. We identified all of the unrelated samples in UK Biobank (N ~ 400,000) and selected a random 5% of the samples. We used these 20,275 samples to create the set of genotypes used for LD clumping. We subsetted these samples out of the UK Biobank data, and kept only high-quality SNPs: imputation info score > 0.9, minor allele frequency > 0.1%, and

Hardy-Weinberg $p > 1 \times 10^{-7}$. Additionally, we used a hardcall threshold (--hard-call-threshold in Plink1.9 [16]) of 0.1. This threshold means the following conversion is applied to the imputed data:

Dosage: 0 - 0.1 → genotype is AA

Dosage: 0.9 - 1.1 → genotype is AB

Dosage: 1.9 - 2.0 → genotype is BB

See Plink1.9 documentation for further details: https://www.cog-genomics.org/plink2/input. After applying this conversion, we additionally removed any SNP with missingness > 0.05.

Using this set of SNPs in 20,275 samples, we performed LD clumping in Plink1.9 [16]. We set genome-wide significance at $p < 5 \times 10^{-9}$, performed clumping in a window of 5Mb, allowing for LD down to $r^2 = 0.05$ and down to a secondary p-value (--clump-p2) of 0.05.

Conditional and joint proximal conditional testing in GCTA

After performing LD clumping, we identified the genomic span of each 'clumped' region. Overlapping regions were collapsed into one (larger) locus. We then added 1kb buffer up- and downstream of the locus boundaries.

Within each locus (i.e., genomic window) we extracted all SNPs from the LD reference panel used for clumping, and again used this data to perform joint and conditional testing using GCTA in order to identify any secondary signals in each locus. We did this using --cojo-slct (http://cnsgenomics.com/software/gcta/GCTA_UserManual_v1.24.pdf), which performs proximal conditional testing when individual-level data is not available for exact conditional testing. Again, we set genome-wide significance (--cojo-p) at $p < 5 \times 10^{-9}$.

Genome-wide association studies and meta-analyses in WHR and BMI

Waist-to-hip ratio (WHR)

As a sensitivity check for our WHRadjBMI meta-analysis, we additionally performed a meta-analysis in the waist-to-hip ratio (WHR) phenotype. The meta-analysis was performed identically to the meta-analysis in WHRadjBMI: genome-wide association testing in UK Biobank, performed in BOLT-LMM, was followed by meta-analysis of the summary-level data with pre-existing data from the GIANT consortium.

Because BMI and WHR are phenotypically correlated, conditioning WHR on BMI (to account for general adiposity) can induce false-positive and false-negative associations due to collider bias. To investigate the extent to which collider bias was affecting our data, we additionally performed a meta-analysis of BMI, following the exact analytic steps as those used to analyze WHRadjBMI and WHR.

Identification of sex-dimorphic SNPs genome-wide

Identifying sex-dimorphic SNPs from the index SNPs identified in our meta-analyses can generate bias around whether the SNP effects will be stronger in men or women (or neither). Index SNPs identified in the combined analysis will be more likely to have similar effects in men and women; index in the

women-only analysis will be more likely to have a stronger effect in women (and the same holds for index SNPs identified in the men-only analysis being more likely to have a stronger effect in men). Given this bias, we additionally identified, genome-wide, all SNPs with evidence for sexual dimorphism ($p_{diff} < 5 \times 10^{-9}$).

We tested all SNPs in our meta-analyses for evidence of sexual dimorphism, and then used the Plink clumping approach to identify those SNPs that were independent from one another. This clumping approach is identical to that described for identifying the index SNPs reported in the main paper (including arguments passed to Plink, provided in the main **Methods** as well as on this paper's GitHub repository); the only difference is that the p-value used for clumping was the p_{diff} (test of sexual dimorphism, as calculated in EasyStrata [19]). Using this approach, we identified 61 sex-dimorphic SNPs, 54 of which had stronger effects in women (see **Methods** for more details on identifying effects stronger in men or women).

Finally, the sexual dimorphism test is as follows:

$$t = \frac{\beta_{females} - \beta_{males}}{\sqrt{se_{females}^2 + se_{males}^2 - 2r * se_{females} * se_{males}}} \quad (1)$$

where *se* is the standard error and *r* is the genome-wide Spearman rank correlation coefficient between SNP effects in females and males. True shared genetic architecture between males and females could inflate the value of r. The value of r across all SNPs in the meta-analysis is 0.023.

Therefore, we recalculated r on a set of ~5M null SNPs with p > 0.5 in the combined analysis, *and* in the women-only analysis *and* in the men-only analysis. We estimated the correlation across the betas in men and women across these SNPs and found r = -0.145.

We then recalculated the sexual dimorphism test for all SNPs in the meta-analysis by first calculating the above t-statistic, and then, assuming that the t-statistic is distributed ~N(0,1) (i.e., approximately z-distributed, as is assumed in EasyStrata), calculated the p-values following:

$$p = 2 * pnorm(-abs(z))$$

Recalculating p_{diff} in this manner only somewhat impacted our results. Of the 61 SNPs we initially found to be sexually dimorphic ($p < 5 \times 10^{-9}$) genome-wide, we found that 48 of them remained significantly sexually dimorphic after adjusting the SNPs used to calculate the Spearman rank correlation coefficient.

References

1. Pulit SL, de With SAJ, de Bakker PIW. Resetting the bar: Statistical significance in whole-genome sequencing-based association studies of global populations. Genet Epidemiol. 2017;41: 145–151.

2. Locke AE, Kahali B, Berndt SI, Justice AE, Pers TH, Day FR, et al. Genetic studies of body mass index yield new insights for obesity biology. Nature. Nature Publishing Group, a division of Macmillan Publishers Limited. All Rights Reserved.; 2015;518: 197–206.

3. Shungin D, Winkler TW, Croteau-Chonka DC, Ferreira T, Locke AE, Mägi R, et al. New genetic loci link adipose and insulin biology to body fat distribution. Nature. 2015;518: 187–196.

4. Cole SR, Platt RW, Schisterman EF, Chu H, Westreich D, Richardson D, et al. Illustrating bias due to conditioning on a collider. Int J Epidemiol. 2010;39: 417–420.

5. Day FR, Loh P-R, Scott RA, Ong KK, Perry JRB. A Robust Example of Collider Bias in a Genetic Association Study. Am J Hum Genet. 2016;98: 392–393.

6. Chu AY, Deng X, Fisher VA, Drong A, Zhang Y, Feitosa MF, et al. Multiethnic genome-wide meta-analysis of ectopic fat depots identifies loci associated with adipocyte development and differentiation. Nat Genet. 2017;49: 125–130.

7. Wood AR, Esko T, Yang J, Vedantam S, Pers TH, Gustafsson S, et al. Defining the role of common variation in the genomic and biological architecture of adult human height. Nat Genet. 2014;46: 1173–1186.

8. Finucane HK, Bulik-Sullivan B, Gusev A, Trynka G, Reshef Y, Loh P-R, et al. Partitioning heritability by functional annotation using genome-wide association summary statistics. Nat Genet. 2015;47: 1228–1235.

9. Auton A, Abecasis GR, Altshuler DM, Durbin RM, Bentley DR, Chakravarti A, et al. A global reference for human genetic variation. Nature. 2015;526: 68–74.

10. Frazer KA, Ballinger DG, Cox DR, Hinds DA, Stuve LL, Gibbs RA, et al. A second generation human haplotype map of over 3.1 million SNPs. Nature. 2007;449: 851–861.

11. Altshuler DM, Gibbs RA, Peltonen L, Dermitzakis E, Schaffner SF, Yu F, et al. Integrating common and rare genetic variation in diverse human populations. Nature. 2010;467: 52–58.

12. McCarthy MI, Abecasis GR, Cardon LR, Goldstein DB, Little J, Ioannidis JPA, et al. Genome-wide association studies for complex traits: consensus, uncertainty and challenges. Nat Rev Genet. 2008;9: 356–369.

13. Bulik-Sullivan BK, Loh P-R, Finucane HK, Ripke S, Yang J, Consortium SWG of TPG, et al. LD Score regression distinguishes confounding from polygenicity in genome-wide association studies. Nat Genet. 2015;47: 291–295.

14. Loh P-R, Tucker G, Bulik-Sullivan BK, Vilhjálmsson BJ, Finucane HK, Salem RM, et al. Efficient Bayesian mixed-model analysis increases association power in large cohorts. Nat Genet. Nature Publishing Group; 2015;47: 284–290.

15. Purcell S, Neale B, Todd-Brown K, Thomas L, Ferreira M a. R, Bender D, et al. PLINK: a tool set for whole-genome association and population-based linkage analyses. Am J Hum Genet. 2007;81: 559–575.

16. Chang CC, Chow CC, Tellier LC, Vattikuti S, Purcell SM, Lee JJ. Second-generation PLINK: rising to the challenge of larger and richer datasets. Gigascience. 2015;4: 1–16.

17. Abecasis GR, Altshuler D, Auton A, Brooks LD, Durbin RM, Gibbs RA, et al. A map of human genome variation from population-scale sequencing. Nature. 2010;467: 1061–1073.

18. Yang J, Lee SH, Goddard ME, Visscher PM. GCTA: A tool for genome-wide complex trait analysis. Am J Hum Genet. The American Society of Human Genetics; 2011;88: 76–82.

19. Winkler TW, Kutalik Z, Gorski M, Lottaz C, Kronenberg F, Heid IM. EasyStrata: evaluation and visualization of stratified genome-wide association meta-analysis data. Bioinformatics. 2015;31: 259–261.
